# Supplementary material for: Interwoven traditions in Bell Beaker metallurgy: Approaching the social value of copper at Bauma del Serrat del Pont (Northeast Iberia)
Source: PLoS One. 2021 Aug 9;16(8):e0255818. doi: 10.1371/journal.pone.0255818 (PMC8352022; doi:10.1371/journal.pone.0255818)
Supplement: S1 File — (PDF) [file pone.0255818.s001.pdf]

S1 File. Metallography and SEM-EDS results

Sample E13

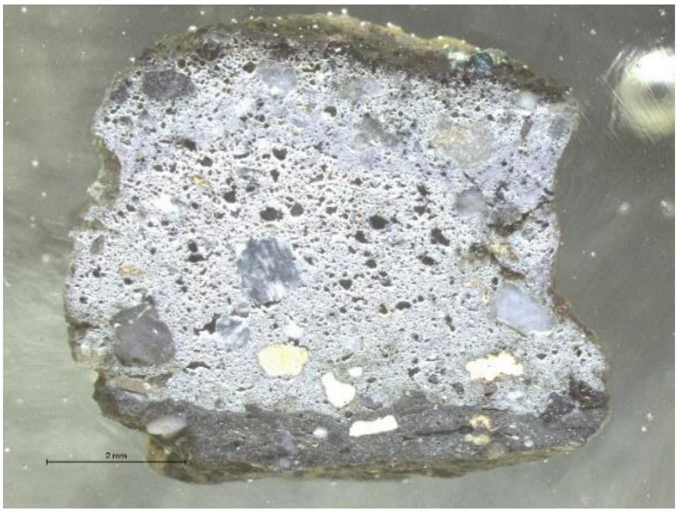

Fig 1. Stereo-microscope reference micrograph of E13.

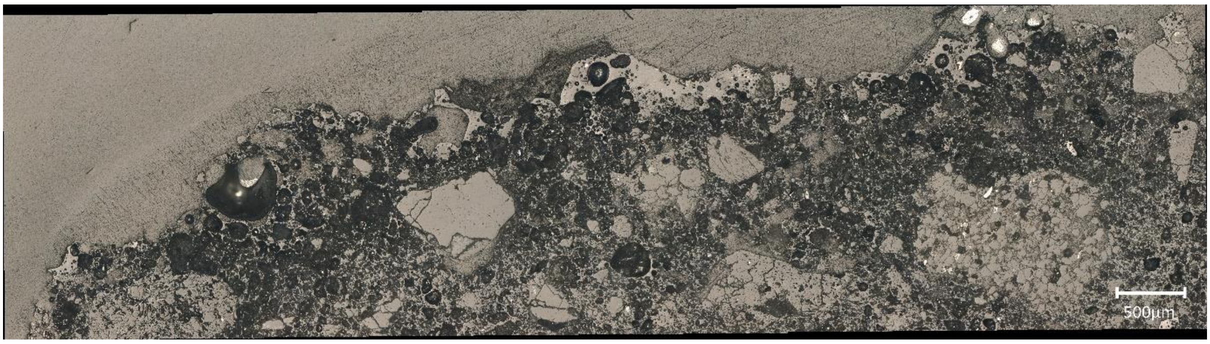

Fig 2. PPL micrograph of E13. General view of slag layer.

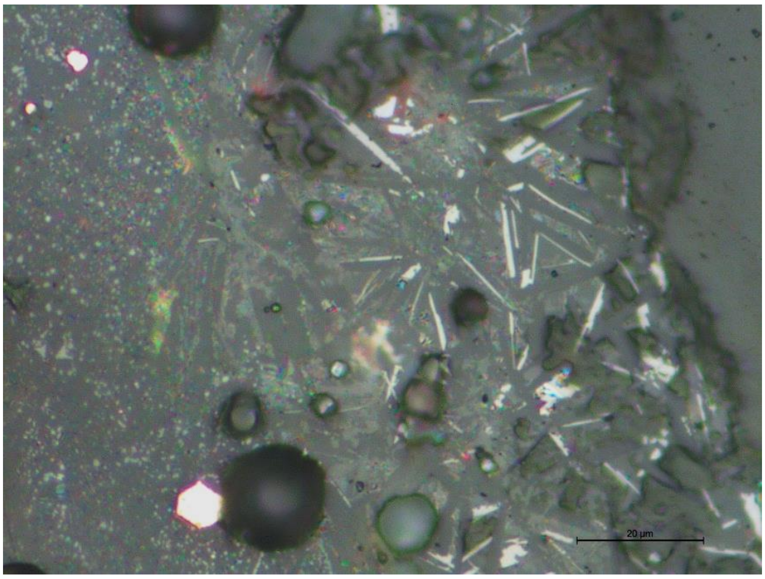

Fig 3. PPL micrograph of E13. Tabular anorthite crystals and elongated delafossite crystals (grey and white respectively, both at the right side of the picture) embedded in a slag matrix rich in small drops of Cu.

Table 1. Bulk ceramic composition of E13.

Description	Na_2O	MgO	Al_2O_3	SiO_2	K_2O	CaO	TiO_2	FeO	Analytical Total
**Close to slag**	0.4	1.3	19.4	59.5	3.2	2.7	0.8	12.7	56.3
**Far from slag**	0.3	1.0	16.6	65.7	2.9	6.2	0.6	6.8	84.2

Table 2. Ceramic matrix composition of E13. (<LOD = below limits of detection).

Description	Na_2O	MgO	Al_2O_3	SiO_2	K_2O	CaO	TiO_2	MnO	FeO	Analytical Total
**Close to slag 1**	0.4	1.1	20.7	63.6	3.6	2.4	0.6	<LOD	7.7	71.5
**Close to slag 2**	0.3	1.4	23.2	56.4	3.4	1.6	0.8	0.5	12.5	84.3
**Close to slag 3**	0.4	1.1	21.4	59.2	4.1	1.6	2.1	<LOD	10.2	100.1
**Far from slag 1**	0.4	1.6	24.7	57.5	3.4	2.3	0.5	<LOD	9.7	108.7
**Far from slag 2**	0.5	1.5	24.6	56.5	4.0	4.2	0.7	<LOD	8.1	110.6
**Far from slag 3**	0.4	1.4	23.6	59.6	3.5	2.1	0.7	<LOD	8.8	102.4

Table 3. Bulk slag composition of E13. (<LOD = below limits of detection).

Description	MgO	Al_2O_3	SiO_2	P_2O_5	K_2O	CaO	TiO_2	MnO	FeO	CuO	PbO	Analytical Total
**Bulk slag 1**	1.3	18.3	50.7	0.4	4.0	2.4	0.7	<LOD	7.3	14.9	<LOD	94.6
**Bulk slag 2**	1.1	13.6	49.3	0.5	4.3	4.6	0.5	0.4	6.5	18.4	0.8	65.9
**Bulk slag 3**	1.1	14.4	59.3	0.8	4.0	2.8	0.7	<LOD	6.2	10.9	<LOD	92.9

Table 4. Glassy matrix composition of E13. (<LOD = below limits of detection).

Description	Na_2O	MgO	Al_2O_3	SiO_2	P_2O_5	K_2O	CaO	TiO_2	FeO	CuO	PbO	Analytical Total
**Glassy matrix 1**	0.4	0.5	10.3	77.4	0.4	4.5	1.3	0.4	3.7	1.2	<LOD	103.0
**Glassy matrix 2**	0.8	0.9	20.4	58.7	0.7	4.7	7.8	0.4	3.4	2.2	<LOD	101.4
**Glassy matrix 3**	<LOD	2.2	19.4	52.0	1.5	5.3	3.9	0.9	8.6	5.7	0.7	98.2
**Glassy matrix 4**	<LOD	1.0	17.5	54.1	0.6	5.4	5.2	0.6	4.7	9.9	1.0	94.6

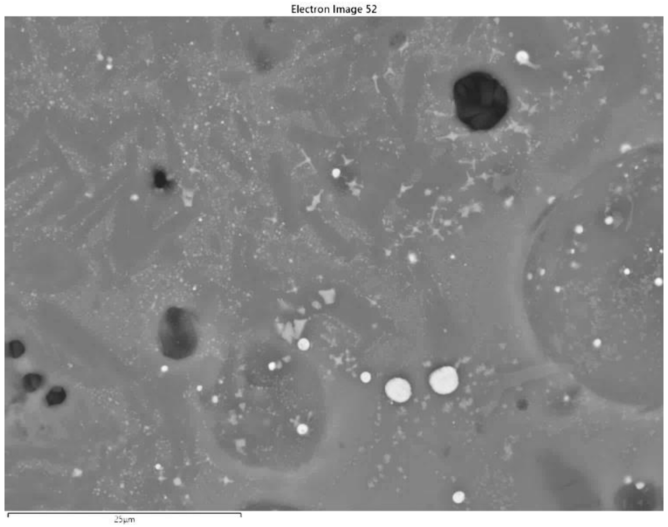

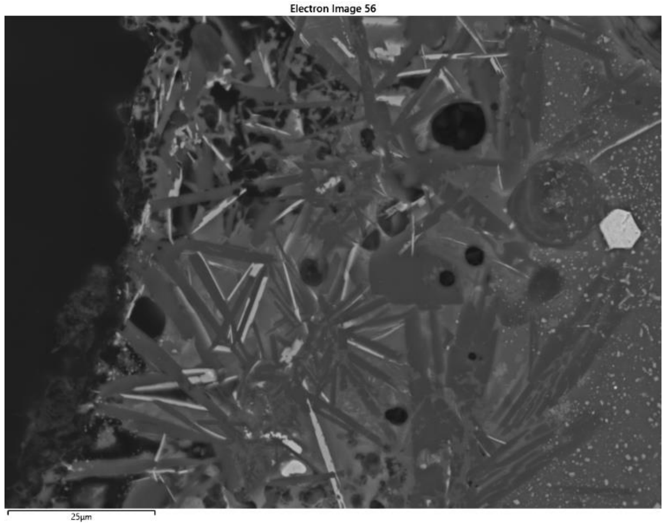

Fig 4 (left). BSE micrograph of E13. Semi-dissolved tabular anorthite crystals (dark grey, S178), Fe alumino-silicate crystals (light grey, S180) and Cu-based prills (white round drops, S176, S177, S181) can be seen (Scale = 25µm).

Fig 5 (right). BSE micrograph of E13. Anorthite crystals (dark grey, S189), delafossite needles (white, S188) and a Cu-based prill (S187) can be seen (Scale = 25µm).

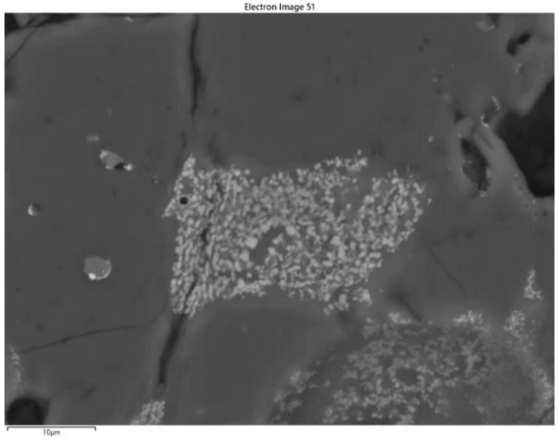

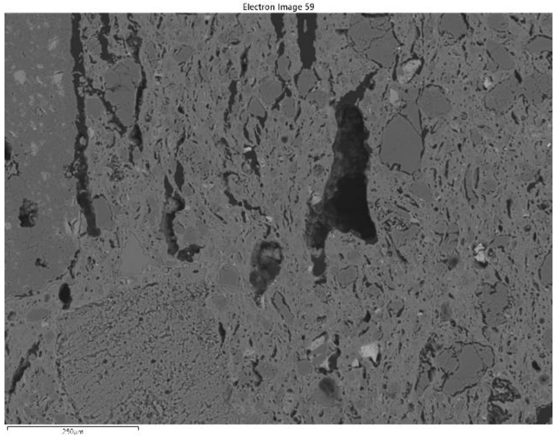

Fig 6 (left). BSE micrograph of E13. Fe-Al-Si mineral semi-dissolved (S173) within a quartz relict (S174) in the glassy matrix (Scale = 10µm).

Fig 7 (right). BSE micrograph of E13. General view of the porosity of the unaltered ceramic paste (Scale = 250µm).

Table 5. Main oxide phases in the slag of E13. (<LOD = below limits of detection).

Description	Spectrum	Na2O	MgO	Al2O3	SiO2	P2O5	K2O	CaO	TiO2	Cr2O3	MnO	FeO	CuO	PbO	Analytical Total
**Fe-Al-Si mineral**	**173**	<LOD	9.9	15.1	32.8	1.2	1.9	1.5	1.7	<LOD	0.3	35.0	0.7	<LOD	98.9
**Quartz inclusion**	**174**	<LOD	<LOD	<LOD	100.0	<LOD	<LOD	<LOD	<LOD	<LOD	<LOD	<LOD	<LOD	<LOD	96.1
**Cu-Si globule**	**177**	<LOD	1.2	9.9	23.2	0.5	1.8	1.3	0.2	<LOD	<LOD	2.3	59.7	<LOD	121.1
**Anorthite crystal (semi-dissolved)**	**178**	0.7	0.6	29.9	47.6	0.4	1.1	15.8	<LOD	<LOD	<LOD	2.7	1.2	<LOD	97.5
**Fe-Al-Si crystal**	**180**	<LOD	3.4	24.3	30.4	0.4	1.6	5.7	1.1	0.4	<LOD	29.0	2.7	0.9	104.8
**Cu-Fe(-Si) needle (delafossite)**	**188**	<LOD	0.9	9.1	12.7	0.5	0.3	4.1	2.3	<LOD	<LOD	32.5	44.5	<LOD	103.9
**Anorthite needle**	**189**	0.3	1.0	27.7	43.6	<LOD	0.7	17.5	<LOD	<LOD	<LOD	3.0	0.8	<LOD	91.5

Table 6. Metallic phases in the slag of E13. (<LOD = below limits of detection).

Description	Spectrum	O	Mg	Al	Si	K	Ca	Fe	Cu	As	Analytical Total
**Prill 1**	**176**	3.6	<LOD	1.9	3.7	0.6	0.4	1.7	87.4	0.8	101.6
**Prill 2**	**181**	6.3	0.5	4.0	7.6	1.0	0.6	1.9	78.0	<LOD	107.2
**Prill 3**	**183**	2.0	<LOD	<LOD	<LOD	<LOD	<LOD	1.0	97.0	<LOD	100.7
**Prill 4**	**185**	1.7	<LOD	<LOD	<LOD	<LOD	<LOD	1.3	97.1	<LOD	103.0
**Prill 5**	**187**	1.3	<LOD	<LOD	<LOD	<LOD	<LOD	1.5	97.3	<LOD	100.8
**Prill 6**	**191**	1.7	<LOD	<LOD	<LOD	<LOD	<LOD	1.6	96.7	<LOD	101.7
**Prill 7**	**192**	6.8	<LOD	3.7	7.7	1.0	<LOD	1.8	79.1	<LOD	107.6

Table 7. Characterisation of non-plastic inclusions of the ceramic paste of E13. (<LOD = below limits of detection).

Description	Spectrum	MgO	Al2O3	SiO2	P2O5	K2O	CaO	TiO2	Cr2O3	MnO	FeO	Analytical Total
**Chromite**	**194**	2.9	8.3	1.4	<LOD	<LOD	0.3	<LOD	63.4	<LOD	23.7	105.2
**Quartz 1**	**195**	<LOD	<LOD	99.7	<LOD	<LOD	<LOD	<LOD	<LOD	<LOD	0.3	103.4
**Quartz 2**	**202**	<LOD	<LOD	100.0	<LOD	<LOD	<LOD	<LOD	<LOD	<LOD	<LOD	105.7
**Fe-Si-Al rock (2 phases)**	**200**	1.4	7.8	82.4	<LOD	1.1	<LOD	<LOD	<LOD	<LOD	7.4	87.7
**Fe-Si mineral (1 phase)**	**201**	0.5	7.4	13.9	0.9	0.7	0.6	0.4	<LOD	<LOD	75.6	99.3
**Fe mineral**	**207**	<LOD	3.5	6.9	1.5	0.3	1.6	<LOD	<LOD	0.6	85.6	78.1

Sample F11

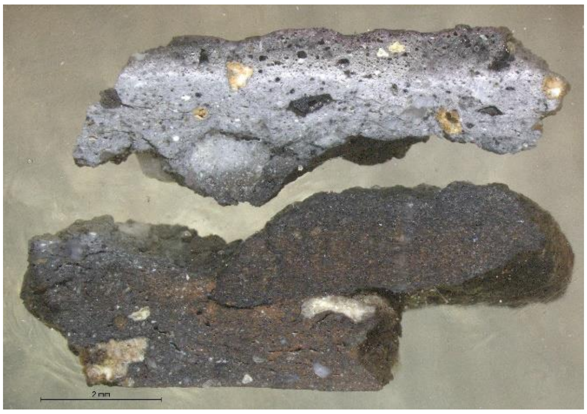

Fig 8. Stereo-microscope reference micrograph of F11.

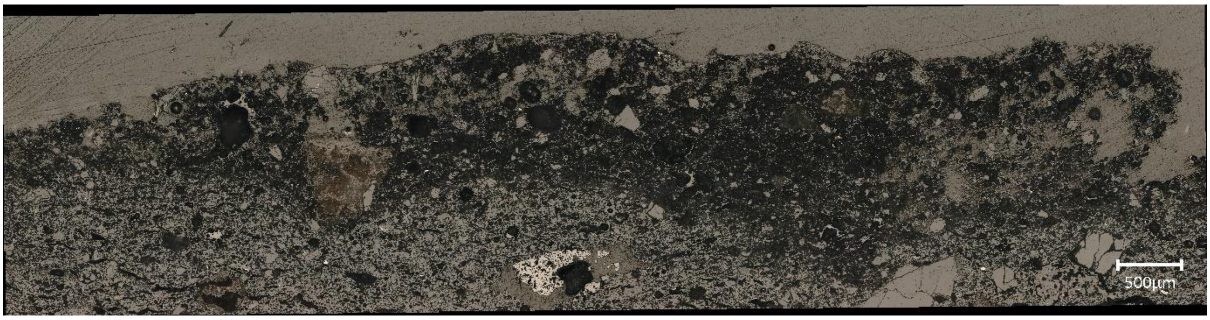

Fig 9. PPL micrograph of F11. General view of slag layer.

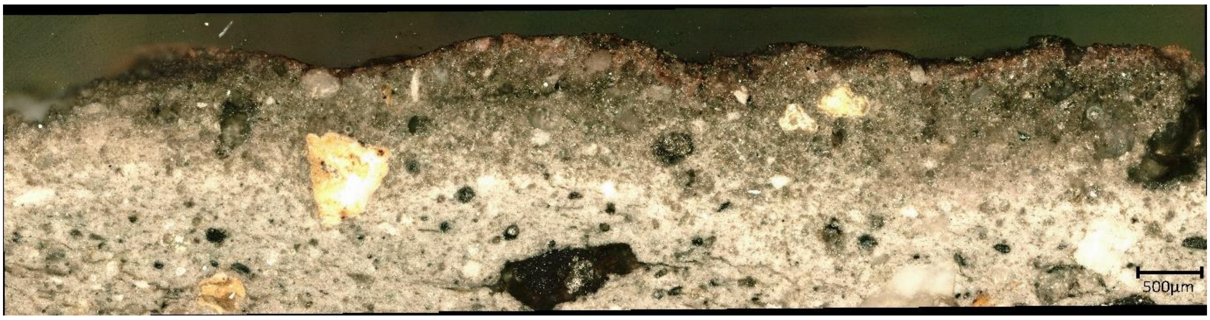

Fig 10. XPL micrograph of F11. General view of slag layer.

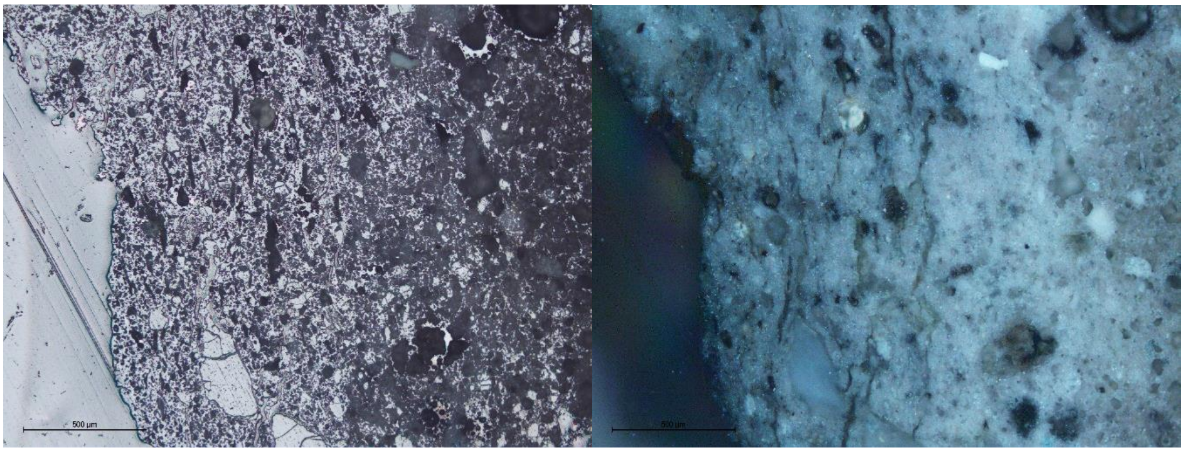

Fig 11. PPL (left) and XPL (right) micrograph of F11 showing the elongated porosity of the unaltered ceramic body.

Table 8. Bulk ceramic composition of F11.

Description	Na2O	MgO	Al2O3	SiO2	K2O	CaO	TiO2	FeO	Analytical Total
**Close to slag**	0.4	1.7	20.4	57.5	4.3	4.0	0.9	10.8	55.1
**Intermediate**	0.7	1.5	19.8	64.3	3.6	2.0	0.7	7.5	77.8
**Far from slag**	0.8	1.3	17.9	69.0	3.4	2.0	0.6	5.1	80.2

Table 9. Ceramic matrix composition of F11. (<LOD = below limits of detection).

Description	Na2O	MgO	Al2O3	SiO2	P2O5	SO3	Cl	K2O	CaO	TiO2	FeO	CuO	Analytical Total
**Close to slag 1**	0.4	2.0	23.4	62.0	<LOD	<LOD	<LOD	4.4	1.1	0.8	5.8	<LOD	80.6
**Close to slag 2**	<LOD	2.5	11.4	24.8	0.6	0.8	0.4	0.5	53.6	<LOD	4.0	1.4	78.9
**Close to slag 3**	0.6	1.8	25.9	53.1	<LOD	<LOD	<LOD	5.0	2.0	1.1	10.6	<LOD	64.9
**Intermediate 1**	0.6	1.6	23.7	61.8	<LOD	<LOD	<LOD	4.4	1.7	0.7	5.5	<LOD	106.9
**Intermediate 2**	0.7	1.7	31.8	52.2	<LOD	<LOD	<LOD	4.4	1.6	0.5	7.0	<LOD	94.0
**Intermediate 3**	<LOD	0.6	8.1	86.7	1.6	<LOD	<LOD	<LOD	0.6	0.2	2.2	<LOD	121.9
**Far from slag 1**	0.7	1.3	29.3	57.4	<LOD	<LOD	<LOD	7.6	0.6	0.5	2.6	<LOD	99.5
**Far from slag 2**	0.8	1.5	23.6	65.8	<LOD	<LOD	<LOD	5.2	0.5	1.4	1.3	<LOD	103.7
**Far from slag 3**	0.8	1.2	23.0	66.2	<LOD	<LOD	<LOD	4.2	1.6	0.2	2.8	<LOD	94.6

Table 10. Bulk slag composition of F11. (<LOD = below limits of detection).

Description	Na2O	MgO	Al2O3	SiO2	P2O5	K2O	CaO	TiO2	FeO	CuO	Analytical Total
**Bulk slag 1**	<LOD	1.8	17.9	43.0	0.4	1.9	1.1	0.8	33.1	<LOD	80.8
**Bulk slag 2**	1.2	2.3	17.1	64.1	<LOD	6.0	2.2	0.4	4.1	2.6	93.4
**Bulk slag 3**	1.1	1.7	20.8	47.7	<LOD	5.4	6.5	0.7	5.4	10.8	80.1

Table 11. Glassy matrix composition of F11. (<LOD = below limits of detection).

Description	Na2O	MgO	Al2O3	SiO2	P2O5	Cl	K2O	CaO	TiO2	FeO	CuO	Analytical Total
**Glassy matrix 1**	0.5	1.2	18.1	47.8	0.6	<LOD	2.1	1.7	1.2	26.7	<LOD	94.4
**Glassy matrix 2**	0.4	0.9	17.3	48.4	0.5	<LOD	1.9	1.7	1.7	27.1	<LOD	93.2
**Glassy matrix 3**	0.4	1.0	16.0	48.6	0.7	<LOD	1.9	1.8	1.3	28.2	<LOD	94.2
**Glassy matrix 4**	1.5	1.5	21.5	55.0	<LOD	<LOD	5.4	7.8	0.5	6.1	0.8	104.0
**Glassy matrix 5**	1.4	1.1	20.6	56.8	0.4	<LOD	5.3	7.5	0.4	5.1	1.5	95.8
**Glassy matrix 6**	1.4	2.4	24.6	54.9	0.5	0.2	6.8	2.5	0.7	4.3	1.8	94.7

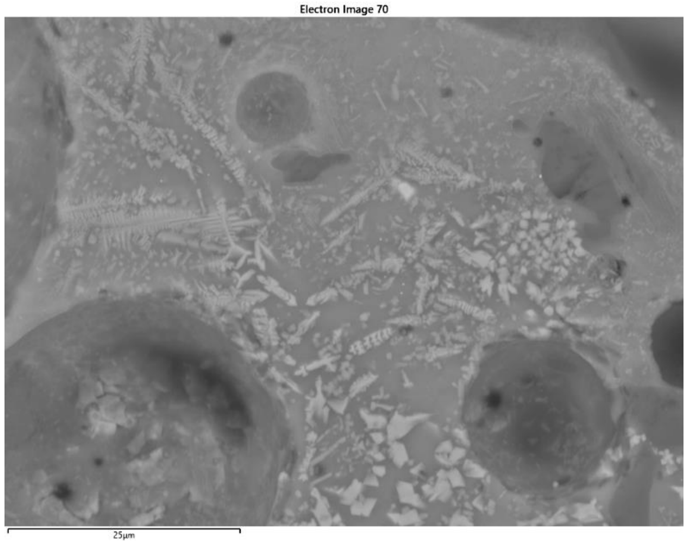

Fig 12. BSE micrograph of F11. Area rich in Fe-Si-Al crystals (S255, S56, S259) clustered together, probably showing molten ceramic inclusions rich in Fe (Scale = 25µm).

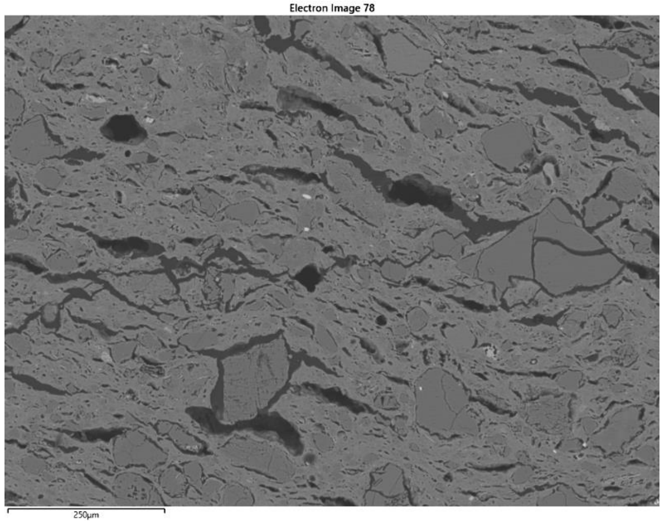

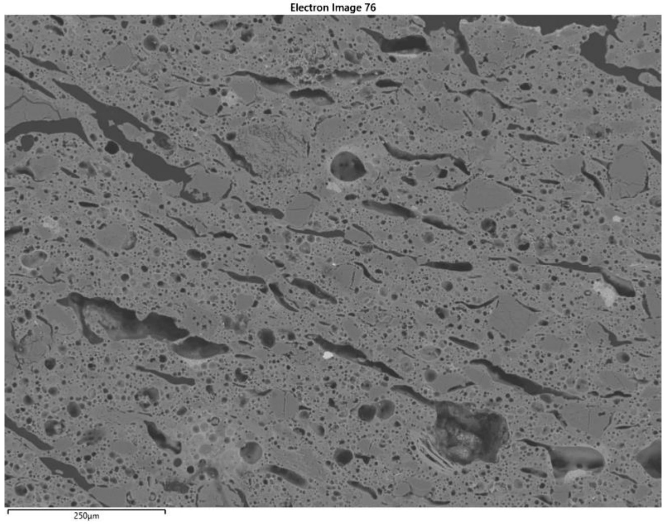

Fig 13 (left). BSE image of F11 showing the porosity of the unaltered ceramic paste rich in non-plastic inclusions (Scale = 250µm).

Fig 14 (right). BSE image of F11 showing the thermochemically altered ceramic matrix of the inner part of the crucible. Note the alteration of the non-plastic inclusions (Scale = 250µm).

Table 12. Main oxide phases in the slag of F11. (<LOD = below limits of detection).

Description	Spectrum	Na2O	MgO	Al2O3	SiO2	P2O5	K2O	CaO	TiO2	FeO	Analytical Total
**Si-Fe-Al crystal 1**	**255**	<LOD	3.4	7.3	40.5	0.5	1.2	0.7	0.4	46.1	101.4
**Si-Fe-Al crystal 2**	**256**	<LOD	5.2	6.3	35.9	0.6	0.8	0.5	0.5	50.2	99.5
**Si-Fe-Al crystal 3**	**259**	0.4	3.4	8.4	38.8	0.6	1.4	0.7	0.3	46.0	96.2

Table 13. Cu-based prills in the slag of F11. (<LOD = below limits of detection).

Description	Spectrum	MgO	Al2O3	SiO2	SO3	K2O	CaO	TiO2	FeO	CuO	As2O3	Analytical Total
**Prill 1 (spot)**	**260**	<LOD	8.6	19.5	0.3	1.5	1.3	<LOD	1.7	63.1	4.1	126.1
**Prill 2 (spot)**	**261**	<LOD	7.9	16.1	<LOD	1.1	0.7	<LOD	1.3	66.5	6.3	127.4
**Peill 3 (spot)**	**262**	0.9	5.8	10.5	<LOD	0.7	0.6	<LOD	1.9	76.4	3.3	119.5
**Prill 4 (spot)**	**263**	<LOD	6.9	24.1	<LOD	1.7	1.2	<LOD	1.3	56.2	8.5	121.8
**Prill 6 (spot)**	**269**	<LOD	9.6	17.0	0.5	1.3	1.6	0.5	1.6	62.5	5.5	116.5
		**O**	**Al**	**Si**	**S**	**K**	**Ca**	**Ti**	**Fe**	**Cu**	**As**	**Analytical Total**
**Prill 5**	**268**	3.4	1.3	1.9	0.2	<LOD	0.2	<LOD	0.8	88.8	3.0	105.5

Table 14. Characterisation of non-plastic inclusions of the ceramic paste of F11. (<LOD = below limits of detection).

Description	Spectrum	Na2O	MgO	Al2O3	SiO2	P2O5	SO3	K2O	CaO	TiO2	MnO	FeO	ZrO2	Analytical Total
**Quartz 1 – semi-dissolved**	**275**	<LOD	<LOD	0.2	99.3	<LOD	<LOD	0.2	0.3	<LOD	<LOD	<LOD	<LOD	99.8
**Quartz 2 – semi-dissolved**	**277**	<LOD	<LOD	4.5	94.2	<LOD	<LOD	1.3	<LOD	<LOD	<LOD	<LOD	<LOD	100.3
**Quartz 3 – semi-dissolved**	**280**	<LOD	<LOD	0.7	96.0	1.6	<LOD	0.1	0.3	<LOD	<LOD	1.3	<LOD	90.6
**Quartz 4**	**284**	<LOD	<LOD	<LOD	97.8	<LOD	<LOD	<LOD	<LOD	1.6	<LOD	0.7	<LOD	94.7
**Quartz 5**	**293**	0.3	0.3	9.9	86.3	<LOD	<LOD	2.4	0.3	<LOD	<LOD	0.5	<LOD	102.2
**Zircon 1**	**279**	<LOD	<LOD	<LOD	31.2	<LOD	<LOD	<LOD	<LOD	<LOD	<LOD	0.9	67.9	96.7
**Zircon 2**	**286**	<LOD	<LOD	<LOD	33.2	<LOD	<LOD	<LOD	<LOD	<LOD	<LOD	1.4	65.5	91.3
**Zircon 3**	**287**	<LOD	<LOD	1.2	32.4	<LOD	<LOD	<LOD	0.6	<LOD	<LOD	1.9	63.9	88.6
**Zircon 4**	**292**	<LOD	<LOD	<LOD	31.4	<LOD	<LOD	<LOD	<LOD	<LOD	<LOD	0.5	68.2	99.5
**Fe-Si-Al mineral**	**283**	<LOD	1.2	14.7	19.8	1.4	<LOD	0.8	1.4	0.3	0.3	60.0	<LOD	109.6
**Fe-Ti-Si-Al mineral**	**285**	1.5	5.3	19.9	25.3	<LOD	<LOD	0.2	1.5	17.7	0.3	28.2	<LOD	107.3
**K-Al-Si mineral**	**294**	0.8	<LOD	18.7	64.5	<LOD	<LOD	16.0	<LOD	<LOD	<LOD	<LOD	<LOD	109.2

Sample F12

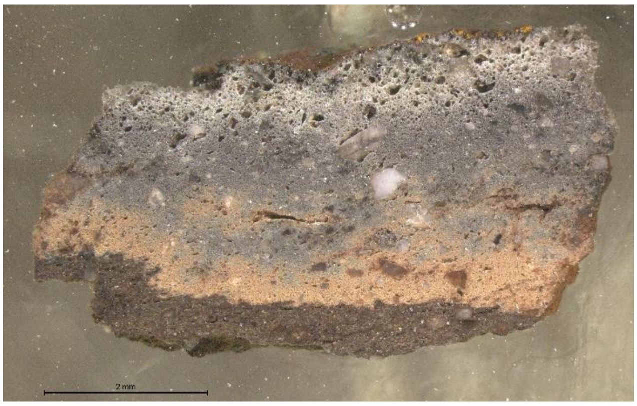

Fig 15. Stereo-microscope reference micrograph of F12.

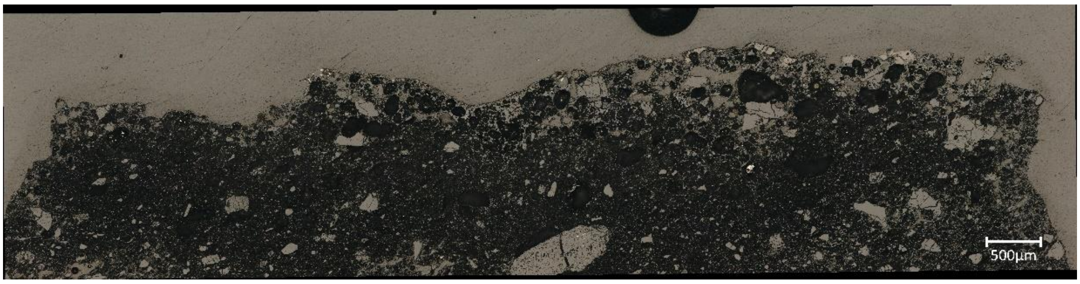

Fig 16. PPL micrograph of F12. General view of slag layer.

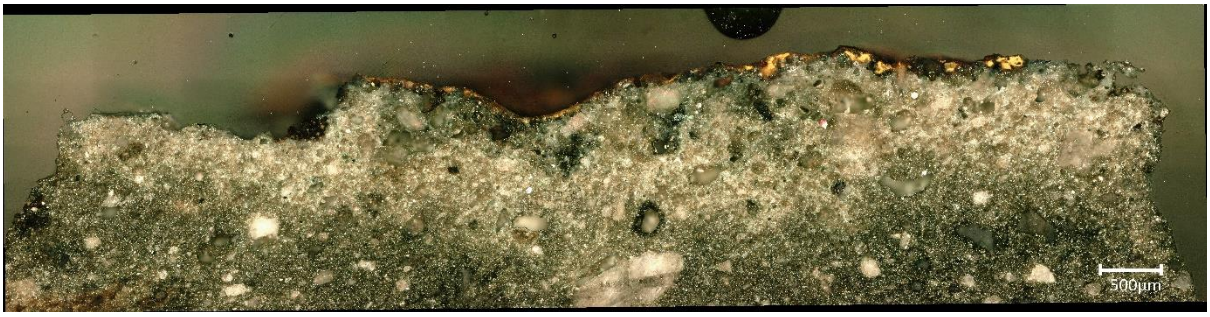

Fig 17. XPL micrograph of F12. General view of slag layer.

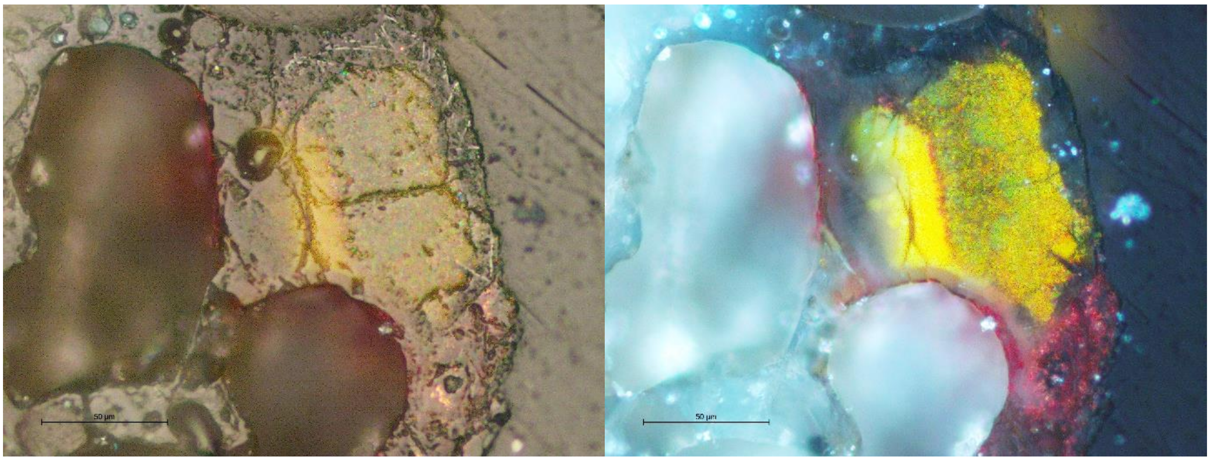

Fig 18. PPL (left) and XPL (right) micrograph of F12 showing a cluster of copper oxide prills (yellow, right side picture) surrounded by delafossite needles (white, top right in left side picture) and anorthite needles (dark grey, top right in left side picture).

Table 15. Bulk ceramic composition of F12. (<LOD = below limits of detection).

Description	Na2O	MgO	Al2O3	SiO2	P2O5	K2O	CaO	TiO2	FeO	Analytical Total
**Close to slag**	0.9	1.4	21.0	56.3	1.7	3.5	3.9	1.3	10.1	59.7
**Intermediate**	0.9	1.1	20.0	63.3	<LOD	5.2	2.3	0.8	6.5	74.2
**Far from slag**	1.5	1.4	19.9	65.2	<LOD	2.9	1.9	0.9	6.5	69.3

Table 16. Ceramic matrix composition of F12. (<LOD = below limits of detection).

Description	Na2O	MgO	Al2O3	SiO2	P2O5	SO3	K2O	CaO	TiO2	FeO	Analytical Total
**Close to slag 1**	0.9	0.6	8.6	24.0	25.5	0.4	2.0	35.0	<LOD	3.1	81.5
**Close to slag 2**	0.5	1.1	15.9	57.3	7.6	<LOD	2.2	10.5	0.4	4.6	86.5
**Close to slag 3**	0.3	1.3	16.3	70.5	<LOD	<LOD	2.9	0.8	0.4	7.5	86.6
**Intermediate 1**	<LOD	1.9	25.6	51.7	<LOD	<LOD	4.9	2.2	0.9	12.9	60.1
**Intermediate 2**	0.4	1.2	18.5	67.5	<LOD	<LOD	4.0	1.9	0.4	6.0	93.3
**Intermediate 3**	0.4	1.5	26.3	60.9	<LOD	<LOD	3.8	1.2	0.6	5.3	75.1
**Far from slag 1**	0.5	2.0	28.9	54.7	<LOD	<LOD	2.9	1.2	1.3	8.5	108.7
**Far from slag 2**	1.2	2.2	26.0	56.6	<LOD	<LOD	2.6	1.3	0.6	9.5	96.6
**Far from slag 3**	0.6	1.9	27.2	58.1	<LOD	<LOD	2.9	1.2	0.6	7.5	101.0

Table 17. Bulk slag composition of F12. (<LOD = below limits of detection).

Description	Na2O	MgO	Al2O3	SiO2	P2O5	K2O	CaO	TiO2	MnO	FeO	CuO	Analytical Total
**Bulk slag 1**	0.9	5.1	14.2	42.2	4.4	2.7	19.9	0.7	<LOD	7.9	2.2	91.9
**Bulk slag 2**	1.2	4.2	13.0	37.5	3.4	3.2	23.7	0.6	0.2	5.9	7.0	93.5
**Bulk slag 3**	1.5	3.6	13.6	41.8	3.0	3.9	23.6	0.5	<LOD	5.2	3.2	89.9

Table 18. Glassy matrix composition of F12. (<LOD = below limits of detection).

Description	Na2O	MgO	Al2O3	SiO2	P2O5	K2O	CaO	TiO2	MnO	FeO	CuO	Analytical Total
**Glassy matrix 1**	2.4	0.8	27.2	48.8	1.0	3.1	12.3	0.3	<LOD	2.6	1.5	102.0
**Glassy matrix 2**	1.3	4.3	13.5	38.9	3.3	2.6	27.6	0.7	0.3	6.0	1.5	97.5
**Glassy matrix 3**	2.3	1.8	11.5	45.1	2.3	3.2	26.6	0.5	<LOD	3.8	3.0	94.0
**Glassy matrix 4**	1.6	7.1	10.0	39.7	1.8	1.4	33.7	<LOD	<LOD	2.4	2.4	99.9
**Glassy matrix 5**	1.4	7.1	9.4	39.3	2.2	1.3	34.6	<LOD	<LOD	2.9	1.9	98.0
**Glassy matrix 6**	2.0	7.4	9.7	39.7	1.8	1.2	33.9	<LOD	<LOD	2.4	1.9	104.5

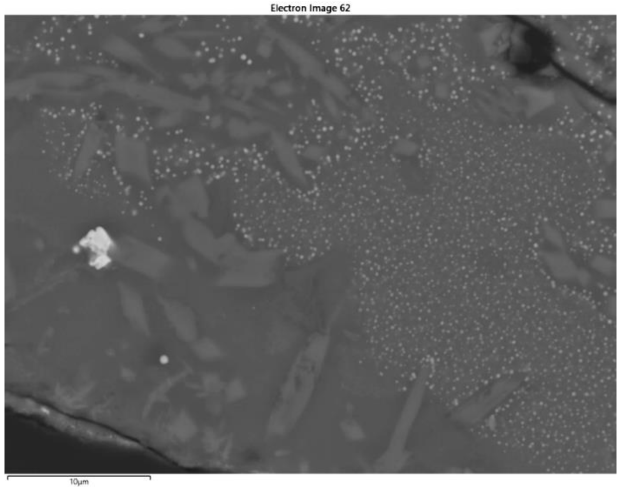

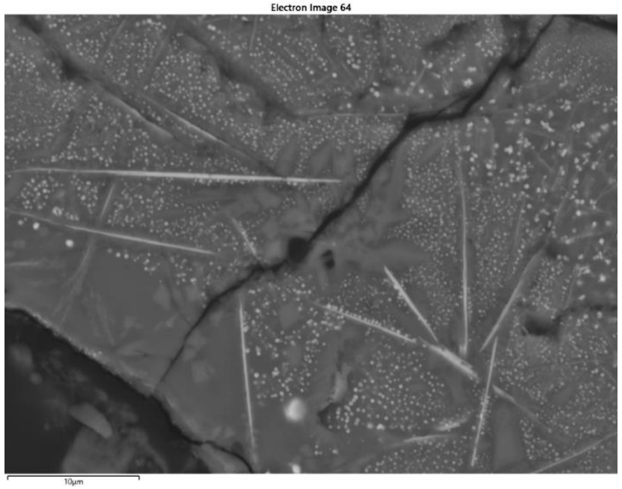

Fig 19 (left). BSE micrograph of F12. Ca-rich crystals (light grey, S217) in a glassy matrix enriched in small Cu-based prills (bright white dots, S216) can be seen. A bigger Cu-based prill is also observed (bright white, S218) (Scale = 10μm).

Fig 20 (right). BSE micrograph of F12. Ca-rich crystal (light grey, S223) and delafossite needles (white, S221) in a glassy matrix enriched in Cu-based small prills (S224) (Scale = 10μm).

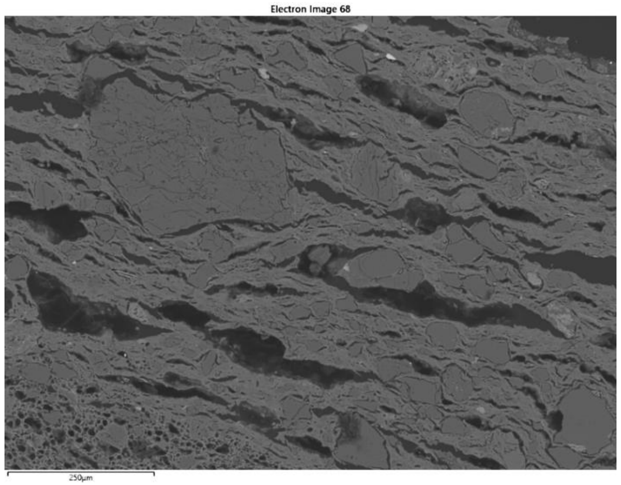

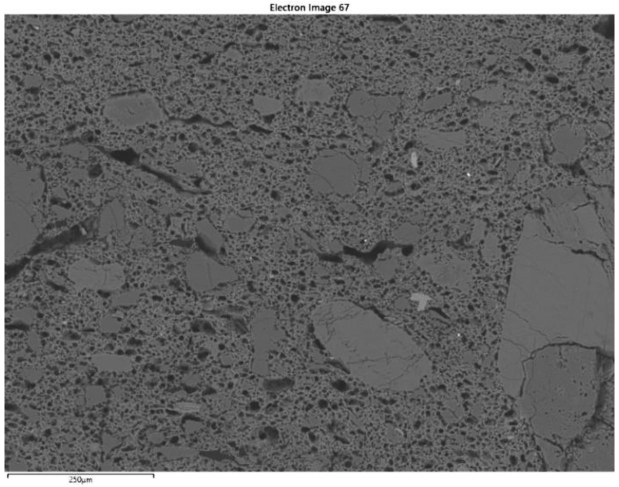

Fig 21 (left). BSE micrograph of F12 showing the porosity of the unaltered ceramic paste rich in non-plastic inclusions (Scale = 250μm).

Fig 22 (right). BSE image of F12 showing the thermochemically altered ceramic matrix of the inner part of the crucible (Scale = 250μm).

Table 19. Main oxide phases in the slag of F12. (<LOD = below limits of detection).

Description	Spectrum	Na2O	MgO	Al2O3	SiO2	P2O5	K2O	CaO	TiO2	MnO	FeO	CuO	Analytical Total
**Glassy matrix with Cu-based prills 1**	**216**	0.9	3.8	12.5	36.4	3.1	3.3	21.8	0.6	<LOD	5.4	12.3	99.1
**Ca-Fe-Si-Al crystal**	**217**	<LOD	7.0	13.2	35.4	2.8	1.0	24.5	1.0	<LOD	12.9	2.0	97.6
**Fe-Cu(-Si) needle (delafossite) (spot)**	**221**	<LOD	2.6	8.5	25.1	1.1	2.0	8.1	1.2	0.3	18.2	33.0	105.9
**Ca-Fe-Si-Al crystal 2**	**223**	0.9	6.4	10.2	42.5	1.9	1.5	23.9	0.7	<LOD	8.1	3.9	99.0
**Glassy matrix with Cu-based prills 2**	**224**	1.8	2.1	10.6	39.2	2.0	3.7	16.8	0.5	<LOD	3.3	20.2	99.1

Table 20. Cu-based prills in the slag of F12. (<LOD = below limits of detection).

Description	Spectrum	O	Mg	Al	Si	P	S	K	Ca	Ti	Fe	Cu	Analytical Total
**Prill 1**	**218**	5.1	0.8	1.7	3.6	0.2	<LOD	0.2	1.9	<LOD	1.1	85.4	109.5
		**Na2O**	**MgO**	**Al2O3**	**SiO2**	**P2O5**	**SO3**	**K2O**	**CaO**	**TiO2**	**FeO**	**CuO**	**Analytical Total**
**Prill 2 (spot)**	**252**	<LOD	2.3	11.7	29.8	2.6	<LOD	2.7	13.0	0.3	3.2	34.5	113.7

Table 21. Characterisation of non-plastic inclusions of the ceramic paste of F12. (<LOD = below limits of detection).

Description	Spectrum	Na2O	MgO	Al2O3	SiO2	P2O5	SO3	K2O	CaO	TiO2	Cr2O3	MnO	FeO	ZrO2	PbO	Analytical Total
**Na-Al-Si mineral 1**	**226**	10.1	<LOD	19.9	68.1	<LOD	<LOD	1.6	<LOD	<LOD	<LOD	<LOD	0.3	<LOD	<LOD	91.8
**Na-Al-Si mineral 2**	**235**	9.4	<LOD	21.0	66.8	<LOD	<LOD	1.9	1.0	<LOD	<LOD	<LOD	<LOD	<LOD	<LOD	98.7
**Na-Al-Si mineral 3**	**240**	8.1	0.6	23.4	64.3	<LOD	<LOD	1.4	0.6	<LOD	<LOD	<LOD	1.5	<LOD	<LOD	85.0
**Zircon 1**	**227**	<LOD	<LOD	3.9	33.6	<LOD	<LOD	0.6	1.7	<LOD	<LOD	<LOD	2.7	57.6	<LOD	71.6
**K-Al-Si mineral 2**	**232**	0.5	18.6	64.3	64.3	<LOD	<LOD	16.7	<LOD	<LOD	<LOD	<LOD	<LOD	<LOD	<LOD	105.3
**Rutile**	**233**	<LOD	0.3	1.5	2.2	<LOD	<LOD	0.2	0.2	92.3	3.3	<LOD	3.3	<LOD	<LOD	109.0
**Pb-Si mineral (spot)**	**234**	<LOD	1.0	9.6	15.1	<LOD	<LOD	1.4	0.8	<LOD	<LOD	<LOD	2.7	<LOD	69.5	131.0
**Ilmenite (spot)**	**243**	<LOD	0.5	0.8	<LOD	<LOD	<LOD	<LOD	<LOD	52.9	<LOD	5.2	40.7	<LOD	<LOD	109.0
**Ti-Si mineral**	**244**	<LOD	<LOD	3.2	20.6	<LOD	<LOD	0.5	<LOD	73.9	<LOD	<LOD	1.2	<LOD	<LOD	116.0
	**Spectrum**			**MgO**	**Al2O3**	**SiO2**	**P2O5**	**K2O**	**CaO**	**FeO**	**SrO**	**La_2O_3**	**Ce_2O_3**	**Pr_2O_3**	**Nd_2O3**	**Analytical Total**
**REE mineral (spot)**	**241**			0.3	26.5	22.2	22.9	1.2	1.4	2.2	1.4	7.3	10.8	1.0	3.1	92.5

Sample G10

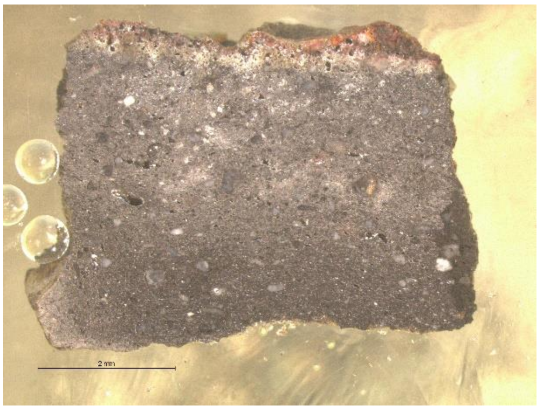

Fig 23. Stereo-microscope reference micrograph of G10.

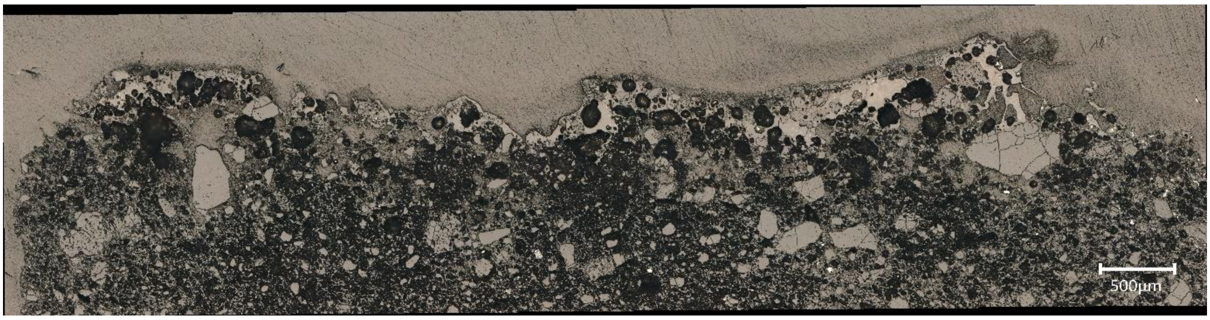

Fig 24. PPL micrograph of G10. General view of slag layer.

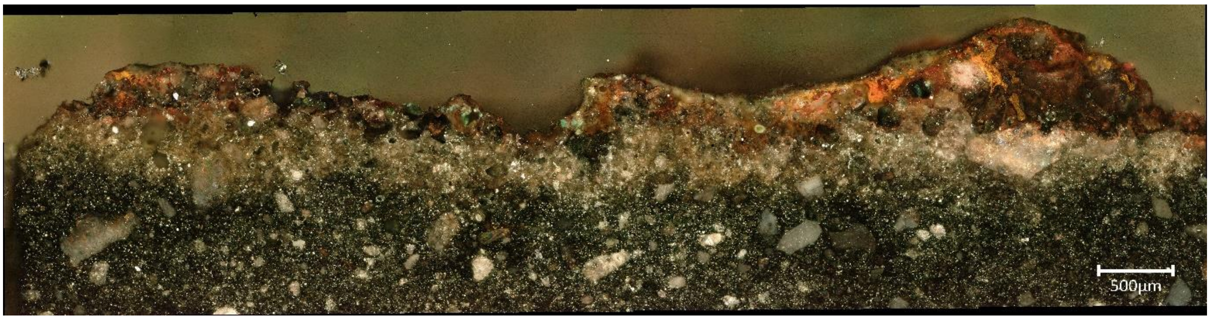

Fig 25. XPL micrograph of G10. General view of slag layer.

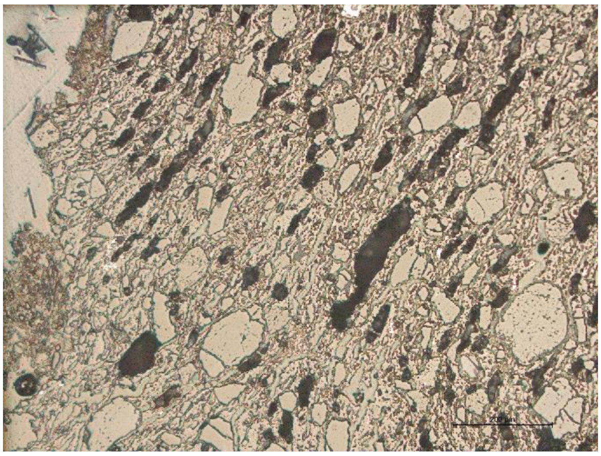

Fig 26. PPL micrograph of G10 showing the elongated porosity of the unaltered part of the ceramic.

Table 22. Bulk ceramic composition of G10. (<LOD = below limits of detection).

Description	Na2O	MgO	Al2O3	SiO2	Cl	K2O	CaO	TiO2	FeO	Analytical Total
**Close to slag**	0.4	0.9	15.9	70.7	0.1	2.7	1.1	1.3	7.0	70.6
**Intermediate**	0.4	0.9	15.5	71.3	<LOD	3.0	1.1	0.8	6.9	73.3
**Far from slag**	0.4	1.0	15.7	71.1	<LOD	2.7	1.0	1.4	6.7	71.7

Table 23. Ceramic matrix composition of G10. (<LOD = below limits of detection).

Description	Na2O	MgO	Al2O3	SiO2	Cl	K2O	CaO	TiO2	MnO	FeO	Analytical Total
**Close to slag**	0.4	1.2	20.3	65.1	0.2	3.3	1.0	0.7	0.3	7.5	73.9
**Intermediate**	0.3	1.1	19.0	65.6	<LOD	3.2	1.1	0.8	<LOD	8.9	64.0
**Far from slag**	0.3	1.0	18.0	67.7	<LOD	3.2	0.8	1.0	<LOD	8.0	80.1

Table 24. Bulk slag composition of G10. (<LOD = below limits of detection).

Description	Na2O	MgO	Al2O3	SiO2	P2O5	K2O	CaO	TiO2	MnO	FeO	CuO	As2O3	Analytical Total
**Bulk slag 1**	0.4	2.6	11.4	40.3	3.6	4.2	24.8	0.7	0.2	5.1	6.7	<LOD	90.8
**Bulk slag 2**	<LOD	2.2	12.7	45.1	1.6	4.0	13.1	0.7	<LOD	5.5	15.1	<LOD	83.2
**Bulk slag 3**	0.7	2.4	15.8	43.1	3.2	2.9	19.1	0.9	<LOD	6.1	3.8	2.1	83.9

Table 25. Glassy matrix composition of G10. (<LOD = below limits of detection).

Description	Na2O	MgO	Al2O3	SiO2	P2O5	K2O	CaO	TiO2	FeO	CuO	As2O3	Analytical Total
**Glassy matrix 1**	0.3	3.2	11.6	40.3	2.1	4.4	28.9	0.7	4.8	3.7	<LOD	101.7
**Glassy matrix 2**	1.0	<LOD	18.8	64.7	<LOD	13.9	<LOD	<LOD	<LOD	1.7	<LOD	102.1
**Glassy matrix 3**	<LOD	2.9	11.0	42.1	3.3	2.0	25.2	0.7	5.1	6.7	1.0	102.5
**Glassy matrix 4**	<LOD	1.2	15.4	48.7	1.2	4.8	17.0	0.7	4.1	5.8	1.0	101.7
**Glassy matrix 5**	<LOD	1.3	12.4	44.2	1.9	2.2	24.2	0.8	5.6	5.7	1.7	103.1
**Glassy matrix 6**	0.7	1.8	13.2	47.2	1.7	2.3	23.5	0.9	5.3	1.7	1.8	104.8
**Glassy matrix 7**	0.5	2.1	12.6	45.2	2.1	2.0	23.0	0.9	7.2	2.7	1.7	102.5
**Glassy matrix 8**	0.5	1.6	13.1	48.8	1.8	3.0	21.0	0.8	6.0	2.4	1.3	105.4

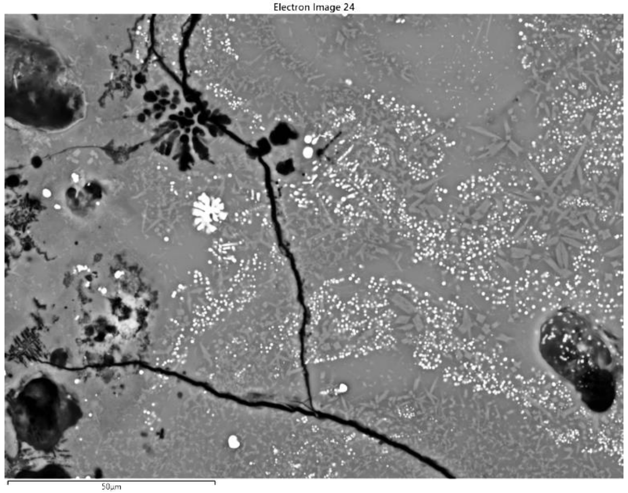

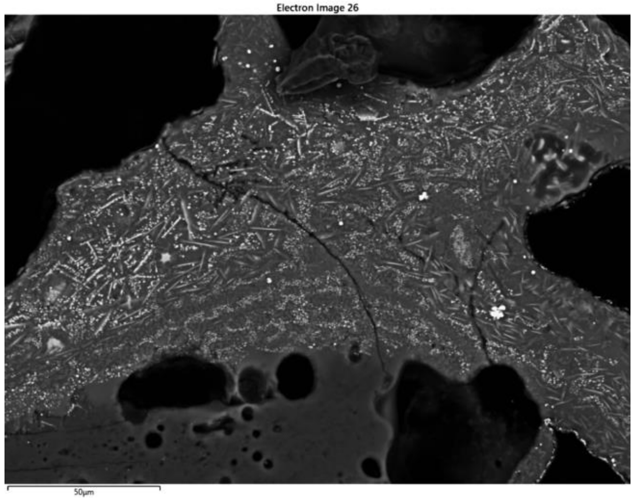

Fig 27 (left). BSE micrograph of G10 showing anorthite crystals (light grey, S66) surrounded by clusters of bright and small Cu-based prills (S67). Bigger bright Cu-based metallic prills can also be seen (S64, S65, S68) (Scale = 50µm).

Fig 28 (right). BSE micrograph of G10 showing a glassy matrix populated by delafossite needles (white, S70), and clusters of small and bright Cu-based prills (S72) (Scale = 50µm).

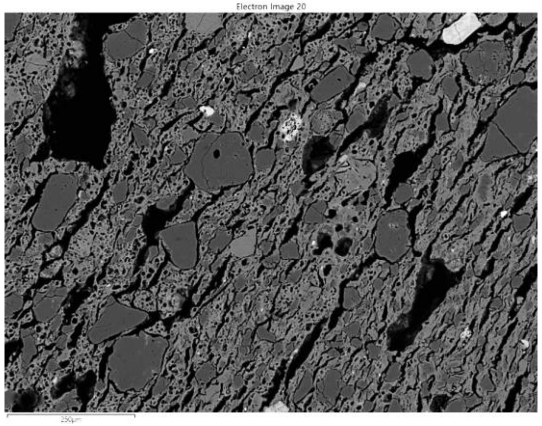

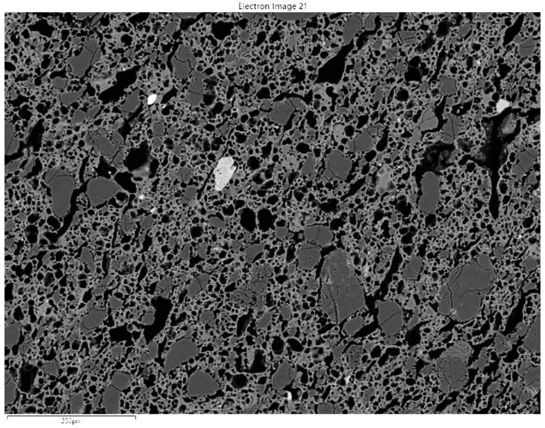

Fig 29 (left). BSE micrograph of G10 showing the porosity of the unaltered ceramic paste rich in non-plastic inclusions (Scale = 250µm).

Fig 30 (right). BSE image of G10 showing the thermochemically altered ceramic matrix of the inner part of the crucible (Scale = 250µm).

Table 26. Main oxide phases in the slag of G10. (<LOD = below limits of detection).

Description	Spectrum	MgO	Al2O3	SiO2	P2O5	K2O	CaO	TiO2	MnO	FeO	CuO	As2O3	Analytical Total
**Anorthite crystal**	**66**	6.64	10.9	42.22	1.82	1.97	22.16	0.97	<LOD	10.74	2.59	<LOD	104.4
**Glassy matrix with Cu-based prills 1**	**67**	1.2	10.3	37.0	1.5	2.9	17.7	0.6	<LOD	3.8	25.0	<LOD	106.5
**Glassy matrix with delafossite and Cu-based prills**	**70**	1.8	11.6	40.1	1.6	3.0	13.2	0.7	0.2	7.0	19.7	1.2	101.4
**Glassy matrix with Cu-based prills 2**	**72**	1.3	12.7	43.7	1.7	4.6	10.3	0.7	<LOD	4.1	20.0	0.9	108.0

Table 27. Cu-based prills in the slag of G10. (<LOD = below limits of detection).

Description	Spectrum	O	Al	Si	P	S	K	Ca	Ti	Fe	Cu	As	Analytical Total
**Prill 1 (spot)**	**64**	11.9	0.9	2.3	<LOD	<LOD	0.3	1.1	<LOD	0.8	82.8	<LOD	104.4
**Prill 2 (spot)**	**65**	1.7	0.3	0.7	<LOD	<LOD	0.2	0.5	<LOD	0.9	95.7	<LOD	102.9
**White mass (spot)**	**68**	12.4	0.7	2.2	0.2	<LOD	0.4	2.3	<LOD	1.2	80.6	<LOD	98.5
**Prill 3**	**80**	1.0	<LOD	<LOD	<LOD	<LOD	<LOD	<LOD	<LOD	0.9	98.1	<LOD	102.0
**Prill 4 (spot)**	**83**	1.3	<LOD	<LOD	<LOD	<LOD	<LOD	<LOD	<LOD	1.0	97.7	<LOD	104.2
**Prill 5 (spot)**	**84**	1.5	<LOD	<LOD	<LOD	<LOD	<LOD	<LOD	<LOD	1.4	97.1	<LOD	100.9
**Prill 6 (spot)**	**85**	3.6	1.7	3.7	<LOD	0.2	0.5	1.3	<LOD	1.2	86.3	1.6	112.4
**Prill 7**	**91**	3.4	1.5	3.7	<LOD	<LOD	0.2	2.1	<LOD	1.4	86.2	1.4	110.5
**Prill 8**	**92**	3.3	1.0	2.4	<LOD	0.2	0.2	1.0	<LOD	0.8	89.2	2.0	108.7
**Prill 9 (spot)**	**93**	13.6	3.1	8.0	0.3	<LOD	0.5	2.5	<LOD	1.2	69.0	1.8	125.8
**Prill 10 (spot)**	**94**	8.4	2.6	6.3	0.2	<LOD	0.4	2.3	<LOD	1.2	77.1	1.5	122.4
**Prill 11 (spot)**	**95**	2.9	0.6	1.9	<LOD	<LOD	0.2	1.3	<LOD	1.1	90.6	1.4	106.9
**Prill 12 (spot)**	**99**	6.0	2.0	5.2	<LOD	<LOD	0.8	0.9	0.1	0.9	83.4	0.7	115.3
**Prill 13 (spot)**	**100**	4.6	1.7	3.9	<LOD	<LOD	0.7	1.1	0.2	1.3	85.8	0.7	111.9

Table 28. Characterisation of non-plastic inclusions of the ceramic paste of G10. (<LOD = below limits of detection).

Description	Spectrum	MgO	Al2O3	SiO2	Cl	K2O	CaO	TiO2	Cr2O3	MnO	FeO	ZrO2	Nb2O5	HfO2	Analytical Total
**Quartz 1**	**48**	0.1	0.2	99.1	<LOD	0.1	<LOD	<LOD	<LOD	<LOD	0.4	<LOD	<LOD	<LOD	102.5
**Quartz 2**	**55**	<LOD	0.3	99.3	<LOD	0.1	<LOD	<LOD	<LOD	<LOD	0.3	<LOD	<LOD	<LOD	99.9
**Quartz 3**	**59**	<LOD	<LOD	100.0	<LOD	<LOD	<LOD	<LOD	<LOD	<LOD	<LOD	<LOD	<LOD	<LOD	104.4
**Fe-Al-Si mineral (spot)**	**49**	1.0	22.1	49.0	0.1	3.1	1.2	1.4	<LOD	<LOD	12.5	9.7	<LOD	<LOD	56.7
**Ilmenite 1**	**50**	<LOD	0.6	1.1	<LOD	0.1	<LOD	65.4	<LOD	6.3	26.6	<LOD	<LOD	<LOD	111.5
**Ilmenite 2 (spot)**	**51**	0.6	0.6	0.5	<LOD	<LOD	<LOD	60.3	<LOD	3.4	34.7	<LOD	<LOD	<LOD	103.9
**Ilmenite 3**	**54**	0.5	5.2	7.3	<LOD	0.4	0.2	74.9	0.5	<LOD	11.0	<LOD	<LOD	<LOD	116.0
**Zircon**	**57**	<LOD	<LOD	30.7	<LOD	<LOD	<LOD	<LOD	<LOD	<LOD	0.3	65.9	1.8	1.4	99.9

Sample G11

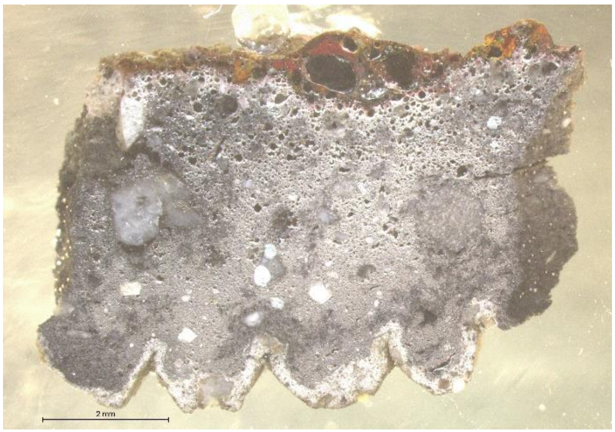

Fig 31. Stereo-microscope reference micrograph of G11.

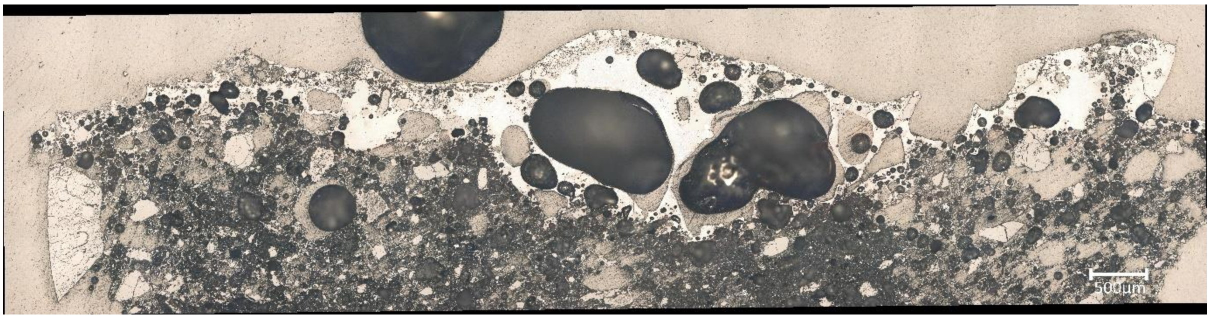

Fig 32. PPL micrograph of G11. General view of slag layer.

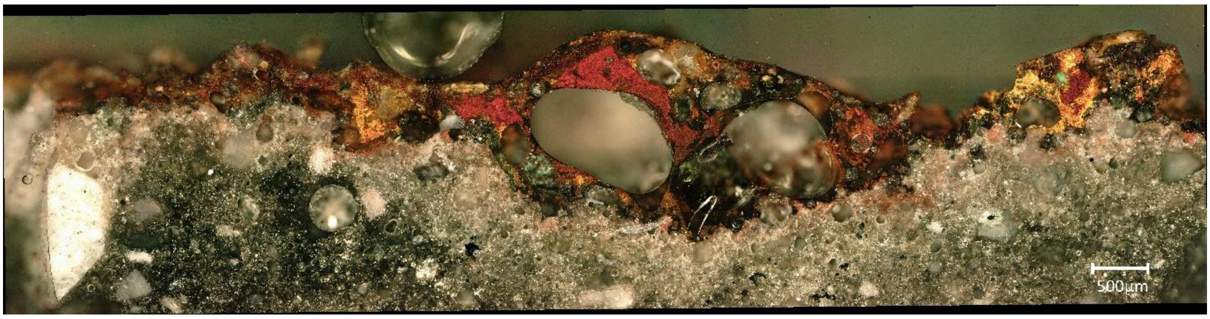

Fig 33. XPL micrograph of G11. General view of slag layer.

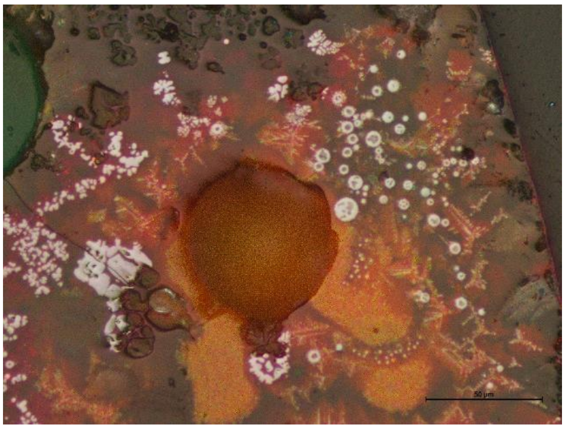

Fig 34. PPL micrograph of G11 showing Cu-based prills of different sizes, clusters of cuprite tinny prills and skeletal cuprite.

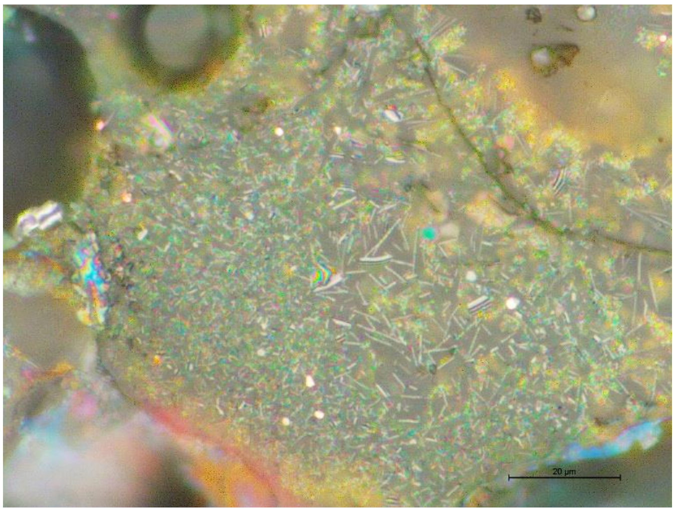

Fig 35. PPL micrograph of sample G11 showing a cluster of elongated delafossite crystals and some small areas rich in cuprite (orangish yellow). Small metallic Cu drops can also be seen (bright yellow).

Table 29. Bulk ceramic composition of G11. (<LOD = below limits of detection).

Description	Na2O	MgO	Al2O3	SiO2	P2O5	K2O	CaO	TiO2	FeO	Analytical Total
**Close to slag**	0.9	1.5	21.4	58.9	1.1	3.3	1.6	1.0	10.5	51.6
**Intermediate**	0.8	1.0	18.4	66.6	<LOD	4.0	1.1	0.8	7.3	65.0
**Far from slag**	0.8	1.1	20.5	63.2	<LOD	4.1	1.2	0.8	8.4	64.4

Table 30. Ceramic matrix composition of G11. (<LOD = below limits of detection).

Description	Na2O	MgO	Al2O3	SiO2	Cl	P2O5	K2O	CaO	TiO2	FeO	Analytical Total
**Ceramic matrix 1**	1.9	<LOD	19.1	65.4	0.1	<LOD	10.3	0.6	<LOD	2.7	87.7
**Ceramic matrix 2**	0.9	1.4	25.0	57.4	<LOD	0.4	4.3	1.4	0.7	8.5	81.1

Table 31. Bulk slag composition of G11.

Description	MgO	Al2O3	SiO2	P2O5	K2O	CaO	TiO2	FeO	CuO	Analytical Total
**Bulk slag 1**	2.5	13.6	45.0	1.7	2.4	15.5	0.5	5.6	13.3	87.6
**Bulk slag 2**	2.1	12.4	41.0	1.3	2.2	11.7	0.5	4.2	24.7	97.8
**Bulk slag 3**	2.3	11.4	39.4	1.3	1.5	10.5	0.4	3.8	29.3	92.1

Table 32. Glassy matrix composition of G11.

Description	MgO	Al2O3	SiO2	P2O5	K2O	CaO	TiO2	FeO	CuO	Analytical Total
**Glassy matrix 1**	3.0	13.4	43.4	1.9	1.7	16.2	0.6	6.9	13.0	100.8
**Glassy matrix 2**	2.8	13.4	43.5	1.9	1.7	15.4	0.7	6.6	14.0	100.1
**Glassy matrix 3**	3.2	13.3	42.9	2.1	2.5	19.0	0.6	5.9	10.7	98.2
**Glassy matrix 4**	2.8	14.1	43.4	1.8	2.7	15.4	0.6	5.6	13.6	95.8
**Glassy matrix 5**	2.4	15.1	45.1	1.6	2.9	11.5	0.7	5.4	15.3	97.4

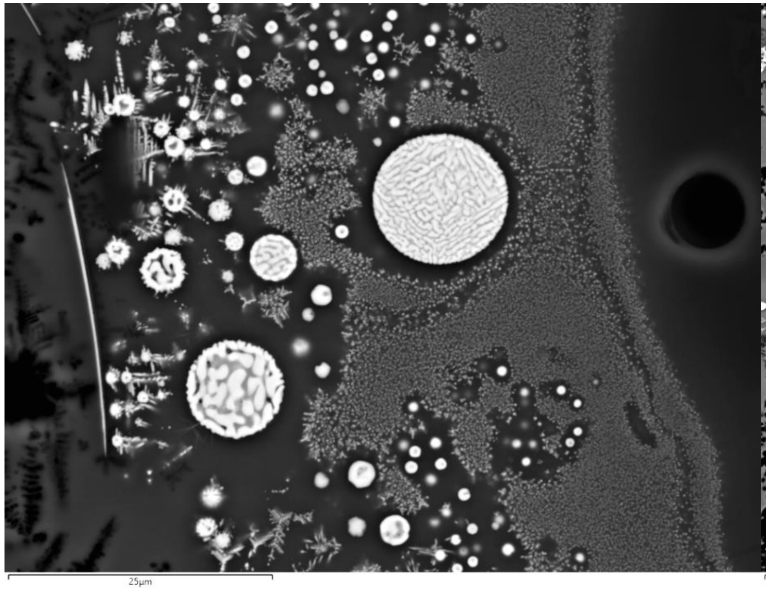

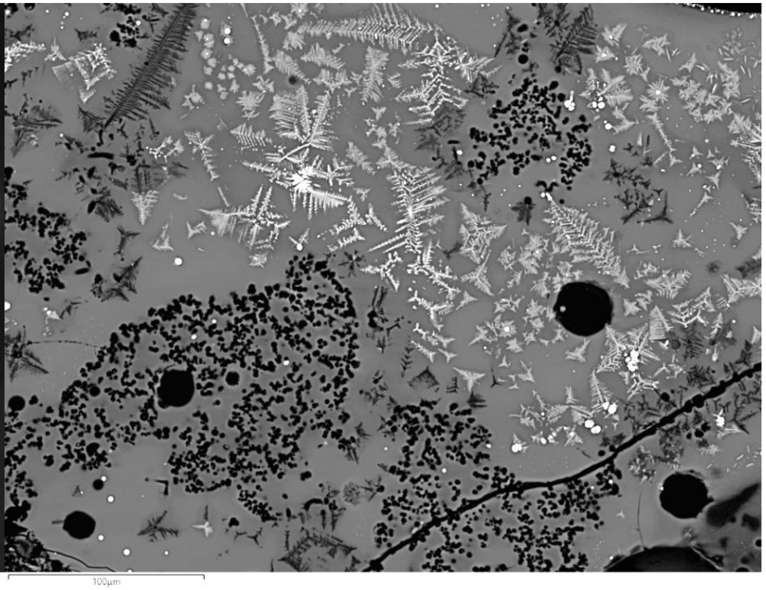

Fig 36 (left). BSE micrograph of G11 showing different biphasic Cu-based prills (S21, S22, S24), an area rich in Cu-based tiny prills (light grey, S25) and a white delafossite needle (S27) (Scale = 25µm).

Fig 37 (right). BSE micrograph of G11 showing clusters of Ca-rich neosilicates (black, S30) and skeletal cuprite (S19) that sometimes emanates out of Cu-based prills (Scale = 100µm).

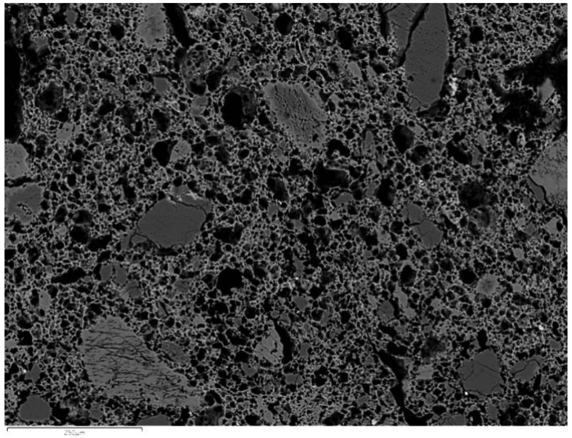

Fig 38. BSE micrograph of G11 showing the thermochemically altered ceramic matrix of the outer part of the vessel, the most far-away surface from the heat source (Scale = 250µm).

Table 33. Main oxide phases in the slag of G11. (<LOD = below limits of detection).

Description	Spectrum	Na2O	MgO	Al2O3	SiO2	P2O5	K2O	CaO	TiO2	Cr2O3	FeO	CuO	Analytical Total
**Delafossite needle (spot)**	**27**	<LOD	1.8	9.8	29.7	0.8	1.5	6.3	1.5	0.2	17.1	31.4	105.1
**Glassy matrix with Cu-based prills**	**25**	<LOD	1.9	12.9	41.3	1.3	3.4	10.6	0.4	<LOD	3.7	24.5	96.1
**Neosilicate rich in Ca (spot)**	**30**	1.8	1.0	19.7	46.8	1.1	2.4	11.7	0.4	<LOD	5.2	9.9	92.3
**Skeletal cuprite (spot)**	**19**	<LOD	1.0	8.3	26.1	0.9	1.8	4.3	0.2	<LOD	1.7	55.7	109.7

Table 34. Cu-based prills in the slag of G11. (<LOD = below limits of detection).

Description	Spectrum	O	Al	Si	P	K	Ca	Ti	Fe	Cu	As	Ag	Sn	Analytical Total
**Prill 1**	**17**	10.7	0.5	1.0	<LOD	<LOD	0.4	<LOD	0.8	86.6	<LOD	<LOD	<LOD	103.2
**Prill 2**	**18**	10.8	0.5	1.2	<LOD	0.2	0.4	<LOD	0.8	86.2	<LOD	<LOD	<LOD	104.4
**Prill 3 – phase grey (spot)**	**21**	24.4	1.1	7.0	<LOD	0.6	1.7	1.8	24.5	38.0	0.4	<LOD	0.6	97.5
**Prill 3 – white phase (spot)**	**22**	12.0	0.8	1.7	<LOD	0.2	0.5	<LOD	1.0	83.9	<LOD	<LOD	<LOD	104.5
**Prill 4 – two phases**	**23**	14.1	0.5	2.1	<LOD	0.2	1.0	0.5	7.4	74.1	<LOD	<LOD	<LOD	98.0
**Prill 5 – two phases**	**24**	13.9	0.2	2.5	<LOD	0.2	0.4	0.5	6.5	75.9	<LOD	<LOD	<LOD	97.8
**Prill 6**	**32**	16.6	4.7	11.5	0.3	1.8	1.5	0.1	1.0	62.1	0.5	<LOD	<LOD	111.5
**Prill 7**	**33**	1.7	<LOD	<LOD	<LOD	<LOD	<LOD	<LOD	0.5	97.8	<LOD	<LOD	<LOD	103.4
**Prill 8**	**34**	1.8	<LOD	<LOD	<LOD	<LOD	<LOD	<LOD	0.4	97.5	<LOD	0.3	<LOD	103.2
**Prill 9**	**35**	1.8	<LOD	<LOD	<LOD	<LOD	<LOD	<LOD	0.5	97.7	<LOD	<LOD	<LOD	101.8
**Prill 10**	**36**	1.6	<LOD	<LOD	<LOD	<LOD	<LOD	<LOD	0.6	97.4	<LOD	0.5	<LOD	102.0
**Prill 11**	**37**	1.8	<LOD	<LOD	<LOD	<LOD	<LOD	<LOD	0.6	97.3	<LOD	0.4	<LOD	103.5

Table 35. Characterisation of non-plastic inclusions of the ceramic paste of G11. (<LOD = below limits of detection).

Description	Spectrum	Na2O	MgO	Al2O3	SiO2	K2O	CaO	TiO2	MnO	FeO	Analytical Total
**Quartz 1**	**38**	<LOD	<LOD	<LOD	99.8	<LOD	<LOD	<LOD	<LOD	0.2	89.8
**Quartz 2**	**40**	<LOD	<LOD	<LOD	99.7	<LOD	<LOD	<LOD	<LOD	0.3	95.4
**Ilmenite inside quartz 2 (spot)**	**41**	<LOD	<LOD	0.2	2.5	<LOD	<LOD	51.3	3.7	42.2	98.1
**Quartz 3**	**42**	<LOD	<LOD	<LOD	99.8	<LOD	<LOD	<LOD	<LOD	0.2	91.9
**Quartz 4**	**44**	<LOD	0.2	1.9	96.7	0.5	<LOD	<LOD	<LOD	0.7	88.4
**Quartz – semi-dissolved**	**45**	<LOD	<LOD	0.7	98.7	0.2	0.4	<LOD	<LOD	0.4	88.8
**K-feldspar – semi-dissolved**	**46**	1.7	<LOD	19.1	65.2	13.2	0.9	<LOD	<LOD	0.9	92.0

Sample H12

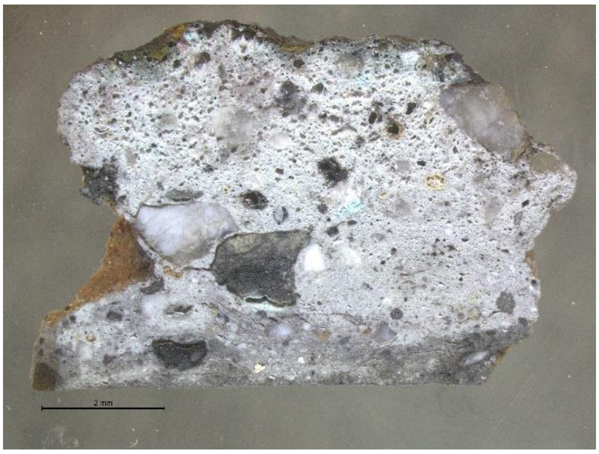

Fig 39. Stereo-microscope reference micrograph of H12.

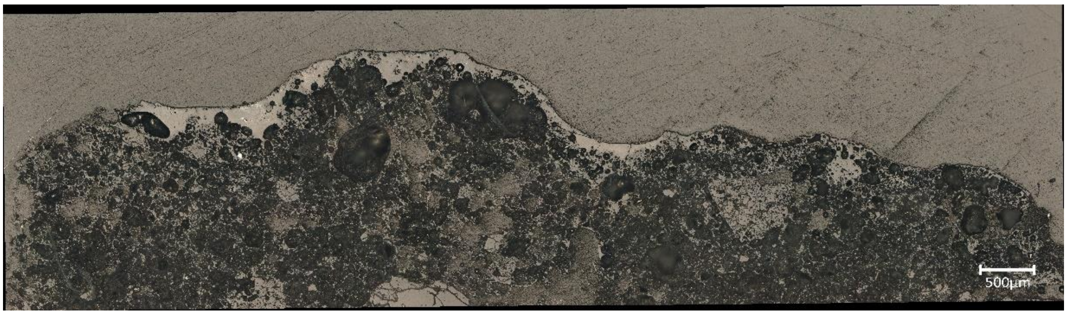

Fig 40. PPL micrograph of H12. General view of slag layer.

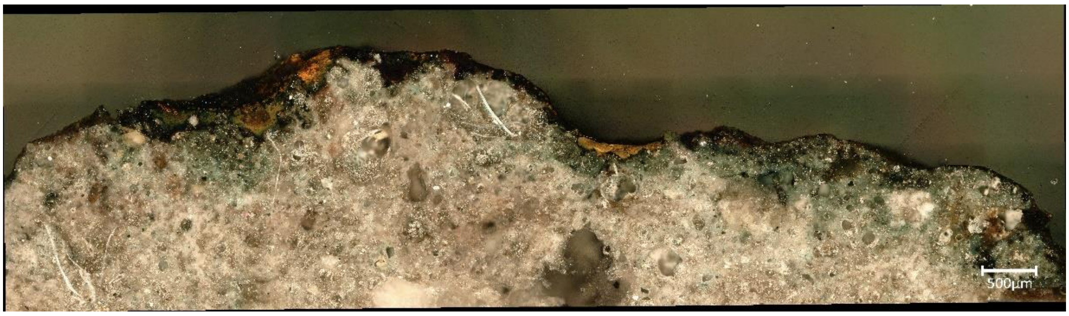

Fig 41. XPL micrograph of H12. General view of slag layer.

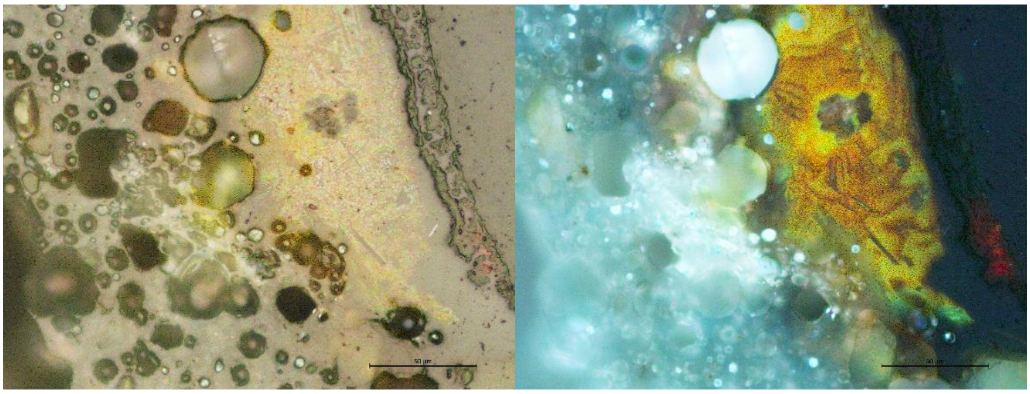

Fig 42. PPL (left) and XPL (right) micrograph of H12. Cluster of cuprite tinny prills (yellow) with anorthite tabular crystals in it (bluish grey) within the glassy layer of the crucible. Potential ore relict rich in CaO.

Table 36. Bulk ceramic composition of H12. (<LOD = below limits of detection).

Description	Na2O	MgO	Al2O3	SiO2	P2O5	Cl	K2O	CaO	TiO2	FeO	Analytical Total
**Far from slag**	0.8	1.1	18.6	65.4	<LOD	0.1	5.9	2.4	0.6	5.2	72.9
**Intermediate**	0.4	2.1	21.5	58.5	0.3	<LOD	4.3	3.2	0.7	8.8	61.3
**Close to slag 1**	0.4	1.4	18.3	59.6	0.3	0.1	3.0	6.7	0.7	9.6	55.3
**Close to slag 2**	0.6	1.8	23.9	55.8	<LOD	<LOD	4.9	2.3	1.0	9.7	49.6

Table 37. Ceramic matrix composition of H12. (<LOD = below limits of detection).

Description	Na2O	MgO	Al2O3	SiO2	P2O5	Cl	K2O	CaO	TiO2	FeO	Analytical Total
**Far from slag 1**	0.7	1.6	22.4	62.7	<LOD	<LOD	4.3	1.9	0.7	5.8	79.7
**Far from slag 2**	0.6	1.6	22.9	61.1	<LOD	<LOD	3.8	2.5	0.8	6.8	96.7
**Far from slag 3**	0.6	1.6	22.3	60.4	<LOD	<LOD	3.4	4.3	0.6	6.9	83.9
**Far from slag 4**	0.6	1.8	24.3	59.3	<LOD	<LOD	4.1	2.8	0.7	6.4	95.8
**Far from slag 5**	0.4	2.1	25.9	56.6	<LOD	<LOD	5.6	1.5	1.4	6.6	84.2
**Far from slag 6**	0.4	1.7	25.0	58.8	<LOD	<LOD	5.1	1.4	0.9	6.7	84.5
**Far from slag 7**	0.5	1.7	26.1	56.9	<LOD	<LOD	5.9	1.6	0.7	6.7	90.7
**Intermediate 1**	0.4	1.7	22.1	59.6	<LOD	<LOD	4.4	3.2	0.7	7.9	62.7
**Intermediate 2**	0.6	1.7	25.2	57.4	<LOD	<LOD	4.7	2.0	0.8	7.7	88.8
**Intermediate 3**	0.6	1.7	21.9	56.5	0.5	<LOD	4.6	4.5	2.2	7.7	73.0
**Intermediate 4**	0.6	1.8	25.2	56.2	<LOD	0.1	5.3	2.0	1.0	8.0	78.0
**Close to slag 1**	0.8	<LOD	19.1	65.8	<LOD	<LOD	12.3	<LOD	<LOD	2.0	94.6
**Close to slag 2**	0.3	1.1	15.7	73.5	<LOD	<LOD	3.6	1.0	0.4	4.5	100.9
**Close to slag 3**	0.3	2.0	15.4	67.0	0.4	<LOD	4.8	1.3	0.8	8.1	65.9

Table 38. Bulk slag composition of H12. (<LOD = below limits of detection).

Description	MgO	Al2O3	SiO2	P2O5	Cl	K2O	CaO	TiO2	FeO	CuO	As2O3	Analytical Total
**Bulk slag 1**	1.6	17.0	46.4	<LOD	3.5	5.3	<LOD	0.6	4.7	19.9	1.0	73.1
**Bulk slag 2**	1.6	14.6	47.4	0.4	<LOD	3.0	8.3	0.5	4.6	18.6	1.0	88.8
**Bulk slag 3**	1.4	16.4	62.1	<LOD	<LOD	4.8	4.1	0.4	5.7	5.1	<LOD	85.5

Table 39. Glassy matrix composition of H12. (<LOD = below limits of detection).

Description	Na2O	MgO	Al2O3	SiO2	P2O5	K2O	CaO	TiO2	MnO	FeO	CuO	As2O3	Analytical Total
**Glassy matrix 1**	<LOD	1.7	13.9	43.1	0.4	3.0	8.7	0.8	<LOD	5.8	21.3	1.3	95.5
**Glassy matrix 2**	<LOD	1.7	15.5	48.6	0.4	4.1	8.6	0.6	<LOD	6.9	13.7	<LOD	91.9
**Glassy matrix 3**	0.5	1.8	27.6	54.2	<LOD	5.9	1.4	0.7	<LOD	6.2	1.7	<LOD	91.6
**Glassy matrix 4**	0.4	1.8	17.3	51.7	<LOD	4.5	6.1	0.7	<LOD	6.6	10.2	0.8	94.0
**Glassy matrix 5**	2.7	13.3	44.7	0.4	2.4	12.8	0.6	0.3	7.0	15.2	<LOD	0.6	96.0

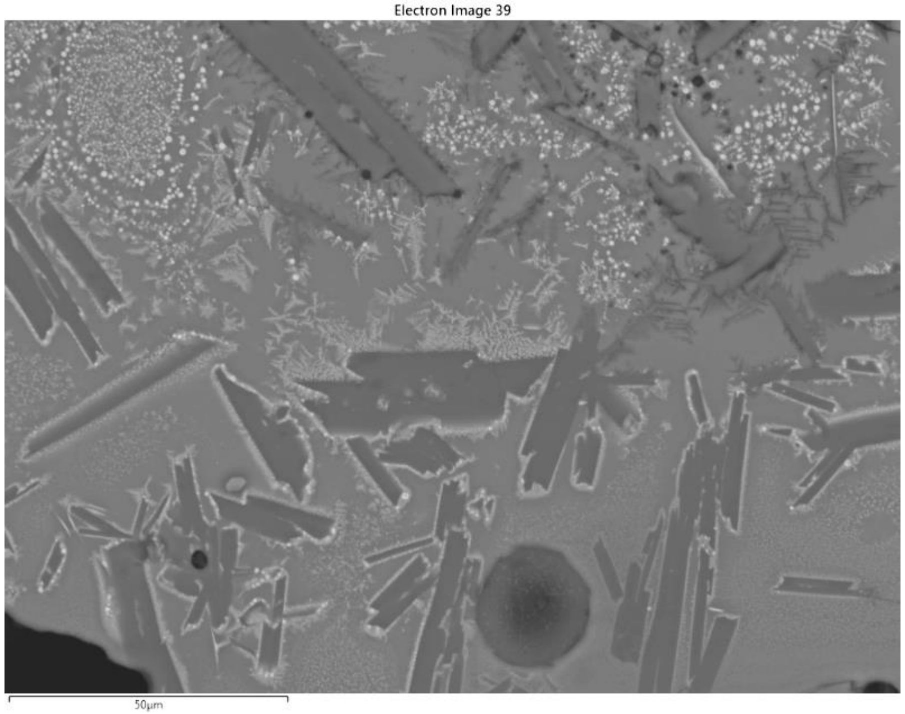

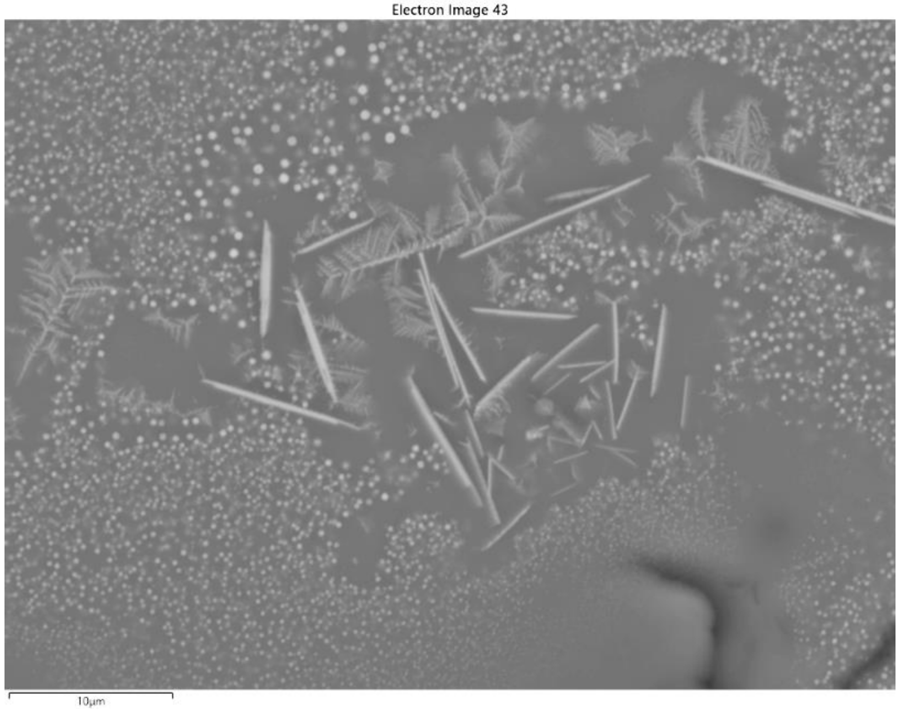

Fig 43 (left). BSE micrograph of H12 showing anorthite crystals (dark grey, S130), skeletal cuprite emanating from them (S132) and a cluster of Cu-based prills at the top right of the picture also rich in CaO (S131) (Scale = 50µm).

Fig 44 (right). BSE micrograph of H12 showing a cluster of delafossite needles (white, S148), skeletal cuprite and Cu-based prills surrounding them (Scale = 10µm).

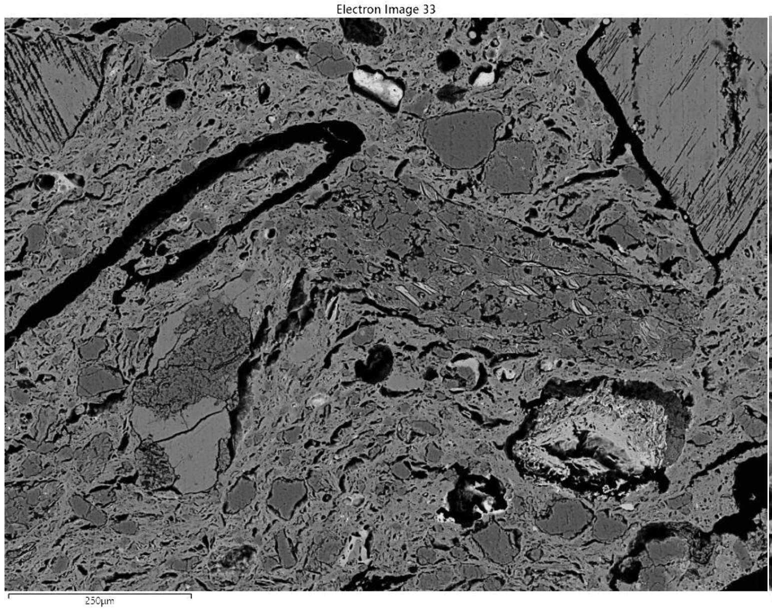

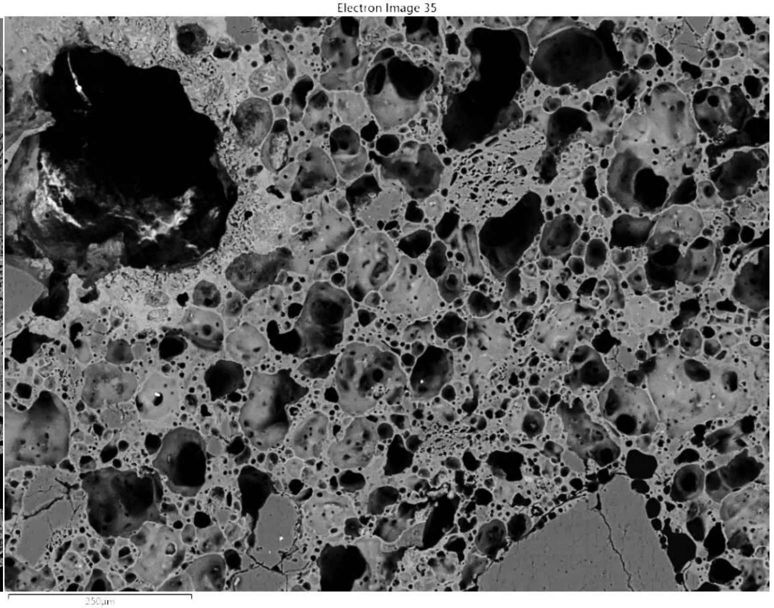

Fig 45 (left). BSE micrograph of H12 showing the porosity of the unaltered ceramic paste rich in non-plastic inclusions (Scale = 250µm).

Fig 46 (right). BSE image of H12 showing the thermochemically altered ceramic matrix of the inner part of the crucible (Scale = 250µm).

Table 40. Main oxide phases in the slag of H12. (<LOD = below limits of detection).

Description	Spectrum	Na2O	MgO	Al2O3	SiO2	P2O5	K2O	CaO	TiO2	FeO	CuO	As2O3	Analytical Total
**Anorthite crystal**	**130**	0.7	0.2	30.7	44.2	<LOD	0.9	15.7	<LOD	1.6	6.0	<LOD	96.9
**Glassy matrix with skeletal cuprite**	**132**	<LOD	1.2	10.8	35.0	<LOD	2.1	8.2	0.4	2.5	38.8	1.1	96.0
**Delafossite**	**148**	<LOD	1.1	8.6	24.7	<LOD	1.2	3.7	2.1	17.9	40.7	<LOD	99.1
**Glassy matrix with Cu-based prills (rich in CaO)**	**131**	<LOD	1.9	11.0	33.2	0.6	1.0	12.4	0.4	6.2	32.2	1.1	95.6

Table 41. Cu-based prills in the slag of H12. (<LOD = below limits of detection).

Description	Spectrum	O	Mg	Al	Si	K	Ca	Ti	Fe	Cu	As	Analytical Total
**Prill 1**	**134**	2.3	<LOD	<LOD	<LOD	<LOD	<LOD	<LOD	0.7	97.0	<LOD	102.2
**Prill 2 (spot)**	**140**	6.3	0.5	1.6	3.5	0.3	0.5	<LOD	1.5	85.9	<LOD	112.2
**Prill 3 (spot)**	**141**	3.8	0.4	1.1	2.7	0.3	0.4	<LOD	1.6	89.7	<LOD	107.8
**Prill 4 (bright phase)**	**151**	11.0	<LOD	<LOD	<LOD	<LOD	<LOD	<LOD	2.9	86.1	<LOD	96.0
**Prill 5 (two phases)**	**152**	16.1	<LOD	0.6	3.3	0.4	0.9	0.7	8.2	68.1	1.8	98.3

Table 42. Characterisation of non-plastic inclusions of the ceramic paste of H12. (<LOD = below limits of detection).

Description	Spectrum	Na2O	MgO	Al2O3	SiO2	P2O5	Cl	K2O	CaO	TiO2	MnO	FeO	ZrO2	HfO2	Analytical Total
**K-Al-Si grey mineral 1**	**103**	2.1	<LOD	18.8	65.0	<LOD	<LOD	14.2	<LOD	<LOD	<LOD	<LOD	<LOD	<LOD	103.1
**K-Al-Si grey mineral 2**	**104**	1.1	<LOD	18.6	64.7	<LOD	0.1	15.2	0.4	<LOD	<LOD	<LOD	<LOD	<LOD	79.6
**Quartz 1**	**105**	<LOD	<LOD	0.3	99.4	<LOD	<LOD	0.1	<LOD	<LOD	<LOD	0.2	<LOD	<LOD	79.3
**Quartz 2**	**154**	<LOD	<LOD	<LOD	100.0	<LOD	<LOD	<LOD	<LOD	<LOD	<LOD	<LOD	<LOD	<LOD	85.4
**Quartz 3**	**157**	<LOD	<LOD	<LOD	100.0	<LOD	<LOD	<LOD	<LOD	<LOD	<LOD	<LOD	<LOD	<LOD	90.5
**Quartz 4**	**162**	<LOD	<LOD	<LOD	100.0	<LOD	<LOD	<LOD	<LOD	<LOD	<LOD	<LOD	<LOD	<LOD	85.3
**Quartz 5**	**165**	<LOD	<LOD	<LOD	100.0	<LOD	<LOD	<LOD	<LOD	<LOD	<LOD	<LOD	<LOD	<LOD	81.1
**Na-Al-Si mineral**	**106**	8.4	<LOD	22.1	65.2	<LOD	0.1	3.2	0.7	<LOD	<LOD	0.2	<LOD	<LOD	74.8
**Fe-Al-Si grey mineral 1**	**108**	0.2	0.7	15.3	69.4	<LOD	<LOD	2.1	1.1	0.6	<LOD	10.7	<LOD	<LOD	64.1
**Al-Si mineral**	**160**	0.8	1.5	35.6	49.6	<LOD	<LOD	8.0	0.6	0.3	<LOD	3.7	<LOD	<LOD	92.0
**Fe-Al-Si grey mineral 2**	**109**	<LOD	0.7	11.0	21.0	1.2	<LOD	0.9	1.4	0.7	0.2	62.9	<LOD	<LOD	100.1
**Zircon (spot)**	**120**	<LOD	<LOD	1.2	34.4	<LOD	<LOD	0.3	<LOD	<LOD	<LOD	1.5	61.1	1.4	112.5
**Rutile**	**159**	<LOD	<LOD	2.0	4.3	<LOD	<LOD	0.6	<LOD	89.7	<LOD	3.5	<LOD	<LOD	65.9
