# Supplementary material for: Interwoven traditions in Bell Beaker metallurgy: Approaching the social value of copper at Bauma del Serrat del Pont (Northeast Iberia)
Source: PLoS One. 2021 Aug 9;16(8):e0255818. doi: 10.1371/journal.pone.0255818 (PMC8352022; doi:10.1371/journal.pone.0255818)
Supplement: S2 File — (PDF) [file pone.0255818.s002.pdf]

S2 File. Summary of the paper in Spanish / Resumen del artículo en castellano

(Este documento es un resumen en castellano del artículo original *Interwoven traditions in Bell Beaker metallurgy: Approaching the social value of copper at Bauma del Serrat del Pont (Northeast Iberia*), publicado en acceso abierto en PLOS ONE en 2021).

Tradiciones entrelazadas en la metalurgia campaniforme: una aproximación al valor social del cobre en la Bauma del Serrat del Pont (nordeste ibérico)

Julia Montes-Landa; Mercedes Murillo-Barroso; Ignacio Montero-Ruiz; Salvador Rovira-Llorens; Marcos Martinón-Torres.

1. Introducción

El nordeste peninsular se encuentra entre dos áreas de conocida tradición metalúrgica: el sur peninsular y el sur de Francia. Durante el III y el II milenio a.n.e., las tres áreas presentan diferencias socio-culturales que derivan en diferentes trayectorias hacia la complejidad social. Dichas trayectorias se reflejan en la distinta importancia social del cobre en estas sociedades.

Este artículo presenta el análisis químico y microestructural de vasijas escorificadas de la Bauma del Serrat del Pont (Tortellà, Girona), como punto de partida para explorar las técnicas metalúrgicas y el suministro de materias primas. Los resultados obtenidos contribuyen a la narrativa de no-linealidad hacia la complejidad social y se muestra que el valor social del cobre influye en el modo de producción y su organización.

2. Caracterización tecnológica y social de la metalurgia temprana del nordeste peninsular: contextualizado la Bauma del Serrat del Pont

2.1 Metalurgia calcolítica en el nordeste: entre dos tradiciones tecnológicas

La metalurgia del sur de Francia y sureste de la península ibérica difieren en tres aspectos respectivamente: (1) el uso de menas sulfídicas *vs.* oxídicas, (2) la reducción en cubeta *vs.* vasijas-horno, (3) el uso o no de recocido en la manufactura de objetos. El conocimiento técnico de la metalurgia en el nordeste se difundió desde el sur de Francia. No obstante, el uso de vasijas-horno, atestado en la Bauma, deriva del sur peninsular.

La Bauma presenta los contextos más tempranos del nordeste con evidencias metalúrgicas (2878-2479 cal. a.n.e.). Los niveles II.3 (Edad del Bronce), II.4, II.5 y III.1 (Calcolíticos) produjeron la mayor parte de hallazgos metalúrgicos. El nivel II.5 es un contexto de producción; el resto, de habitación. Se han recuperado 65 fragmentos de vasijas campaniformes (decoradas y sin decorar) usadas en actividades metalúrgicas, junto con toberas, residuos de fundición y algunos objetos metálicos [1]. Los análisis composicionales (ED-XRF) de estos materiales sugieren que la Bauma es el primer lugar de la península ibérica donde se ha documentado producción de bronce a través de la reducción de menas polimetálicas (Cu-Sn), y es posible encontrar minerales polimetálicos en los alrededores (minas de Les Ferreres y Can Manera). Estos análisis también confirmaron el procesado de cobre en estas vasijas. Nuestro trabajo analítico se centra en clarificar (1) si las vasijas de la Bauma se usaron para reducir o fundir cobre, (2) el tipo de menas usadas (oxídicas *vs.* sulfídicas), (3) su procedencia, (4) la relevancia del uso de vasijas decoradas y (5) si estas vasijas son hechas *ad hoc* para emplearse como crisoles.

2.2. El valor social del cobre calcolítico en el mediterráneo occidental

El valor social del cobre en el sur de la península ibérica durante el Calcolítico (*ca.* 3200-2200 cal. a.n.e.) era reducido. Los objetos de cobre (herramientas o herramientas-arma) se depositaban tanto en contextos habitacionales como funerarios, pero no en las tumbas más ricas. Su bajo valor social puede estar asociado al amplio acceso a menas de cobre y al carácter doméstico de esta tecnología. En el nordeste, las comunidades del Neolítico-Final/Calcolítico no reducían cobre, pero poseían adornos (cobre y oro) y herramientas-arma

metálicas. Dichos objetos se depositan en tumbas comunales sin relación con individuos específicos. El mayor papel simbólico del cobre en las comunidades del nordeste puede derivarse de la ausencia de conocimiento técnico para producirlo. En el sur de Francia, pese a producir cobre, su valor social se corresponde con el del nordeste. Solamente en el distrito de Cabrières la producción metálica se asocia a cambios sociales, como los nuevos patrones de hábitat que facilitaban la organización de la producción.

La Edad del Bronce (2250-1550 cal. a.n.e.) en el sureste peninsular supone el desarrollo de elites (El Argar), lo que se correlaciona con la proliferación de ornamentos metálicos (cobre, plata, oro, bronce) y de armas como símbolos de estatus. En el nordeste, alrededor de 2700 a.n.e., emerge el fenómeno campaniforme. Estas comunidades inhuman a un menor número de personas que sus antecesoras y éstas son individualizadas dentro de las tumbas comunales. Los grupos campaniformes producían herramientas y armas metálicas que aparecen depositadas, con evidencias de uso prolongado, en contextos funerarios relacionadas con individuos específicos. Esto muestra una función tanto utilitaria como posiblemente identitaria. La mayor importancia aparente del metal para las comunidades campaniformes que para sus antecesoras, puede relacionarse con el comienzo incipiente de la estratificación social. Sin embargo, un total desarrollo de poder eliticio en esta área no ocurrirá hasta la Edad del Hierro; la producción de cobre se mantiene en una escala reducida durante siglos. Finalmente, en el sur de Francia, pese a los cambios observados en Cabrières, la producción y consumo de metal se reduce en el Languedoc durante el final del III milenio a.n.e. y la producción se transfiere al área de Hautes-Alpes hasta el II milenio a.n.e. Esto se puede relacionar con ciertos cambios sociales en el norte de Italia.

3. Evidencias de metalurgia de crisol en el nordeste peninsular

Las evidencias de minería durante el III milenio a.n.e se concentran en los distritos mineros del Montsant (incluyendo las minas de la Solana del Bepo y la Turquesa) y del Molar-Bellmunt-Falset. Se han encontrado evidencias de metalurgia extractiva en la Cova Joan d'Ós (Tartareu), la Cova del Frare (Matadepera), la Cova Freda de Montsant (Collbató), Vapor Gorina (Sabadell), la Balma del Duc (Montblanc), la Cova del Buldó (Montblanc), la Cova Cartanyà (Vilavert), la Cova de l'Heura (Ulldemolins), la Cova de Porta Lloret (Siurana) y la Cova Josefina d'Escornalbou (Ruidecanyes).

Los materiales encontrados y sus contextos denotan una metalurgia de cobre rudimentaria, doméstica y esporádica. No existe especialización; se lleva a cabo en hábitats temporales en cueva (salvo en Vapor Gorina), lo que contrasta con los contextos abiertos del sur de Iberia y Francia. En estas cuevas también se realizaban inhumaciones, pero dichos enterramientos no se relacionan con los contextos de producción-hábitat (excepto en la Cova del Buldó). El nivel II.5 de la Bauma y el contexto de la Cova de l'Heura destacan por tratarse de áreas dedicadas exclusivamente a la producción de metal.

4. Materiales analizados y sus contextos arqueológicos

Se han analizado siete fragmentos de vasijas campaniformes metalúrgicas escorificadas. También se publican los resultados de unas metalografías realizadas por S. Rovira años atrás en tres crisoles más. Tres de las diez vasijas estudiadas presentan decoraciones incisas. Todas las vasijas provienen de cinco niveles diferentes datados por ^{14}C. Entre los niveles muestreados hay diferentes contextos de habitación y un contexto de producción metalúrgica (nivel II.5). Todas las muestras analizadas pertenecen al horizonte Calcolítico Campaniforme del III milenio a.n.e. La información relacionada con los contextos arqueológicos se puede encontrar en Alcalde *et al.* [1–4] y Soriano [5].

5. Métodos

Tras los análisis de fluorescencia de rayos X (pXRF) en 32 fragmentos de vasijas metalúrgicas, seis fragmentos (H12, E13, G11, F12, F11 y G10) se muestrearon y prepararon para su análisis bajo microscopia óptica y electrónica de barrido (SEM-EDS). También se realizaron análisis de isótopos de plomo (MC-ICP-MS) en cinco muestras (H12, E13, E11, F11 y G10).

6. Resultados

6.1. Caracterización de las cerámicas técnicas

La misma arcilla fue usada para la manufactura de todas las vasijas metalúrgicas: un alumino-silicato con FeO, K_2O y CaO y presencia menor de MgO, Na_2O y TiO_2. Las inclusiones minerales en la cerámica son diversas (cuarzos, feldespatos, aluminosilicatos de Fe y minerales de Ti y Zr), de variado tamaño y formas angulares o subangulares. G10 y H12 presentan inclusiones orgánicas. Probablemente, las inclusiones minerales y orgánicas son componentes naturales de la arcilla.

6.2. Caracterización de las operaciones metalúrgicas

Gran parte de la capa escorificada es cerámica fundida. Se observan diferencias con respecto a la cerámica en las cantidades de MgO, P_2O_5, CaO y CuO. Las capas de escoria de G10, G11 y F12 son más gruesas y están enriquecidas en CaO, MgO y P_2O_5. H12 tiene una capa de escoria gruesa pero menor enriquecimiento en CaO. El enriquecimiento de CaO y MgO puede indicar el uso de minerales con ganga calcítica o dolomítica. El enriquecimiento en P_2O_5 está relacionado con el combustible utilizado o con la ganga. E13 y F11 tienen unas capas de escoria más fina; no están tan enriquecidas en CaO, pero tienen un ligero enriquecimiento de K_2O con respecto a la cerámica, posiblemente derivado del combustible utilizado.

6.2.1. Operaciones metalúrgicas con cargas ricas en Ca

F12, G10, G11 y H12 representan operaciones de reducción de menas de cobre ricas en Ca (calcita/dolomita). Presentan un enriquecimiento general en CaO y MgO, y neosilicatos (frecuentemente anortita) ricos en estos elementos. Todas las muestras contienen delafosita. G11 y H12 contienen microestructuras que pueden relacionarse con relictos de mena. Los altos niveles de FeO de algunos neosilicatos y áreas de la escoria se relacionan con la descomposición de algunos minerales de hierro de la pasta cerámica. Las inclusiones metálicas analizadas son de cobre, con impurezas de Fe (G11, G10, H12), As (G11, G10, H12), Ag y Sn (G11), en algunos casos. Las metalografías previamente tomadas por S. Rovira (PA6326, PA6325 y PA6327) muestran características similares a las nuevas muestras analizadas, pudiéndolas integrar en el mismo tipo de operaciones.

6.2.2. Operaciones metalúrgicas con cargas pobres en Ca

E13 y F11 no contienen evidencias contundentes de reducción, pero es probable que representen esta práctica. E13 contiene aluminosilicatos de Fe secundarios, delafosita y anortita. Su presencia es coherente con operaciones de reducción, pero nunca aparecen agrupados, por lo que no se pueden relacionar con relictos de mena. E13 presenta exsoluciones de cuprita alrededor de gotas metálicas que podrían indicar una re-oxidación. El Pb presente en la matriz vítrea denota que la carga era rica en este elemento. F11 contiene una capa de escoria enriquecida en FeO, que deriva, posiblemente, de la descomposición de minerales de Fe de la pasta cerámica. Las inclusiones metálicas/oxidadas analizadas en ambas muestras son de cobre con pequeñas impurezas de Fe y As.

6.3. Isótopos de plomo

H12 se puede relacionar con la mina de la Solana del Bepo y G10 con la de la Turquesa. Esto último es coherente con la presencia de As en esta muestra, pese a que la ratio $^{207}Pb/^{206}Pb$ se aleja un poco. Se pueden encontrar minerales ricos en Ca en el entorno de estas minas. F11 se puede relacionar con la mina de Les Ferreres, que no cuenta con minerales de Ca en su entorno inmediato. E13 y E11 no concuerdan con las mineralizaciones caracterizadas del nordeste, los Pirineos y Francia. Sus valores siguen la misma tendencia que otras muestras del nordeste, por lo que sería posible relacionarlas con otros recursos regionales no caracterizados por el momento.

7. Discusión y conclusiones

En la Bauma se utilizaron cinco menas, una de la mina de Les Ferreres (a 18km de la Bauma), otras dos de las minas de la Solana del Bepo y la Turquesa respectivamente (a 200km ambas), una cuarta no caracterizada pero posiblemente de la región, y una quinta polimetálica (Cu-Sn) caracterizada en un estudio anterior, probablemente de las minas de Les Ferreres o Can Manera.

Los crisoles analizados se utilizaron para reducir cobre de acuerdo con la tradición ibérica: uso de vasijas metalúrgicas, menas oxídicas y condiciones reductoras moderadas. Pese a la conexión tecnológica con el sur de

Iberia, el nordeste es una esfera cultural separada. No está claro que las cerámicas no decoradas se hicieran con propósitos metalúrgicos. Dichas cerámicas están hechas con la misma arcilla que las cerámicas comunes decoradas, que se reutilizaron para producir metal. Las inclusiones orgánicas y minerales son componentes naturales de la arcilla.

El cobre, en el Calcolítico, era valorado por sus características utilitarias. Esto se refleja en la organización de la producción, y en la manufactura de herramientas-arma. La demanda de cobre era generalmente baja y la producción de carácter doméstico. El nivel II.5 refleja un momento puntual de mayor demanda. Esta adaptabilidad de la producción es consistente con una consideración utilitaria del cobre, ya que un uso funerario/simbólico haría difícil justificar episodios como el del nivel II.5.

El cobre tenía un incipiente valor social: objetos muy usados se depositan en contextos funerarios, algo que se puede relacionar con la falta de evidencias de reciclado. La amplia disponibilidad de minerales de cobre permitía producir más metal y que los objetos adquirieran un papel simbólico en las tumbas.

La explotación de varias menas contemporáneamente se da en diferentes niveles de ocupación. Un cambio de función del yacimiento (doméstica *vs.* producción) no implicaba la reorganización del trabajo más allá de multiplicar las operaciones. Existía una gran versatilidad para adquirir minerales, lo que sugiere un conocimiento de los minerales locales y un mantenimiento de redes regionales. Esta flexibilidad aseguraría el acceso a los recursos necesarios, facilitando el desarrollo de una metalurgia de carácter principalmente utilitario. Los recursos minerales mencionados fueron explotados por diferentes comunidades calcolíticas a la vez.

Durante el II milenio a.n.e., en el sur de la Península Ibérica, se desarrolla el poder eliticio, contribuyendo a incrementar el papel simbólico del metal. Al tiempo, la producción de metal en el Languedoc desaparece. En el nordeste, el proceso de estratificación social se extiende en el tiempo y el cobre compite con otras materias primas en los ámbitos simbólico y utilitario. Ciertos cambios en el valor del metal se observan alrededor de 1600 a.n.e., pero no es hasta la Edad del Hierro I (750/650-550 a.n.e.) cuando se desarrollan jefaturas en el valle del Segre-Cinca. De esta manera, el sur de Iberia, el nordeste y el sur de Francia muestran trayectorias diferentes hacia la complejidad social, en las que el papel del cobre difiere. Es fundamental atender a factores específicos sociales, políticos y medioambientales para explicar los cambios socio-tecnológico a lo largo del tiempo. Este artículo demuestra que es posible contribuir a esta narrativa a través del análisis de restos de producción.

Bibliografía (se remite al lector/a al artículo original para una lista completa)

1. Alcalde G, Molist M, Montero I, Planagumà L, Tled A. Producciones metalúrgicas en el nordeste de la Península Ibérica durante el III milenio cal. a.C.: El Taller de la Bauma del Serrat del Pont (Tortellà, Girona). Trabajos de Prehistoria. 1998;55: 81–100.

2. Alcalde G, Molist M, Saña M, Toledo A. Procès d'ocupació de la Bauma del Serrat del Pont (La Garrotxa) entre el 2900 y el 1450 cal AC. Museu Comarcal de la Garrotxa; 1997.

3. Alcalde G, Molist M, Toledo A. La Bauma del Serrat del Pont. Memòria de les campanyes d'excavació 1991-1994. 1994.

4. Alcalde G, Molist M, Toledo I Mur A, Caravaca J, Codina D. La Bauma del Serrat del Pont, Tortellà, La Garrotxa: un taller de metal·lúrgia del coure d'ara fa 4000 anys. Annals de l'Institut d'Estudis Gironins. 1994;XXXIII: 43–48.

5. Soriano Llopis I. Producción Metalúrgica Prehistórica en el Nordeste de la Península Ibérica (Mediados del IV-II Milenio cal. ANE). Aportaciones Cronoculturales, Tecnológicas y Funcionales. Vol.I. PhD thesis, Universitat Autònoma de Barcelona. 2010.
