# Supplementary material for: Interwoven traditions in Bell Beaker metallurgy: Approaching the social value of copper at Bauma del Serrat del Pont (Northeast Iberia)
Source: PLoS One. 2021 Aug 9;16(8):e0255818. doi: 10.1371/journal.pone.0255818 (PMC8352022; doi:10.1371/journal.pone.0255818)
Supplement: S3 File — (PDF) [file pone.0255818.s003.pdf]

S3 File. Summary of the paper in Catalan / Resum de l'article en català

(Aquest document és un resum en català de l'article original *Interwoven traditions in Bell Beaker metallurgy: Approaching the social value of copper at Bauma del Serrat del Pont (Northeast Iberia*), publicat en accés obert a PLOS ONE al 2021*)

** Trad.: Marc Gener-Moret*

Tradicions entrellaçades en la metal·lúrgia campaniforme: una aproximació al valor social del coure a la Bauma del Serrat del Pont (nord-est ibèric)

Julia Montes-Landa; Mercedes Murillo-Barroso; Ignacio Montero-Ruiz; Salvador Rovira-Llorens; Marcos Martinón-Torres.

1. Introducció

El nord-est peninsular es troba entre dues àrees de coneguda tradició metal·lúrgica: el sud peninsular i el sud de França. Durant el III i el II mil·lennis a.n.e. les tres àrees presenten diferències sòcio-culturals que deriven en diferents trajectòries envers la complexitat social. Dites trajectòries es reflecteixen en la diferent importància social del coure en aquestes societats.

Aquest article presenta l'anàlisi químic i microestructural de vasos escorificats de la Bauma del Serrat del Pont (Tortellà, Girona) com a punt de partença per a explorar les tècniques metal·lúrgiques i el subministrament de matèries primeres. Els resultats obtinguts contribueixen a la narrativa de no-linealitat cap a la complexitat social. Es mostra que el valor social del coure influeix en el mode de producció i al seva organització.

2. Caracterització tecnològica i social de la metal·lúrgia primerenca del nord-est peninsular: contextualitzant la Bauma del Serrat del Pont

2.1 Metal·lúrgia calcolítica en el nord-est: entre dues tradicions tecnològiques

La metal·lúrgia del sud de França i el sud-est de la península ibèrica difereixen en tres aspectes respectivament: (1) l'ús de menes sulfídiques *vs.* oxídiques, (2) la reducció en cubeta *vs.* en vas-forn, (3) l'ús o no de recuit en la manufactura d'objectes. El coneixement tècnic de la metal·lúrgia al nord-est es va difondre des del sud de França. No obstant això, l'ús de vasos-forn, atestat a la Bauma, deriva del sud peninsular.

La Bauma presenta els contextos més primerencs del nord-est amb evidències metal·lúrgiques (2878-2479 cal. a.n.e.). Els nivells II.3 (Edat del Bronze), II.4, II.5 i III.1 (Calcolítics) van produir la major part de troballes metal·lúrgiques. El nivell II.5 és un context de producció; la resta, d'habitació. S'han recuperat 65 fragments de vasos campaniformes (decorats i sense decorar) fets servir en activitats metal·lúrgiques, juntament amb toveres, residus de fosa i alguns objectes metàl·lics [1]. Les anàlisis composicionals (ED-XRF) d'aquests materials suggereixen que la Bauma és el primer lloc de la península ibèrica on s'ha documentat producció de bronze mitjançant la reducció de menes polimetàliques (Cu-Sn), és possible trobar minerals polimetàl·lics als voltants (mines de les Ferreres i Can Manera). Aquestes anàlisis també van confirmar el processat de coure en aquests vasos. El nostre treball analític es centra en aclarir (1) si els vasos de la Bauma es van fer servir per reduir o fondre coure, (2) el tipus de menes usades (oxídiques *vs.* sulfídiques), (3) la seva procedència, (4) la rellevància de l' ús de vasos decorats i (5) si aquests vasos són fabricats *ad hoc* per emprar-se com a gresols.

2.2 El valor social del coure calcolític en el mediterrani occidental

El valor social del coure al sud de la península ibèrica durant el Calcolític (*ca.* 3200-2200 cal. a.n.e.) era reduït. Els objectes de coure (eines o eines-arma) es dipositaven tant en contextos habitacionals com funeraris, però no en les tombes més riques. El seu baix valor social pot estar associat a l'ampli accés a menes de coure i el caràcter domèstic d'aquesta tecnologia. Al nord-est, les comunitats del Neolític-Final/Calcolític no reduïen coure, però posseïen ornaments (coure i or) i eines-arma metàl·liques. Aquests objectes es dipositen en

tombes comunals sense relació amb individus específics. El major paper simbòlic del coure en les comunitats del nord-est pot derivar-se de l'absència de coneixement tècnic per produir-lo. Al sud de França, tot i produir coure, el seu valor social es correspon amb el del nord-est. Només al districte de Cabrières la producció metàl·lica s'associa a canvis socials, com els nous patrons d'hàbitat que facilitaven l'organització de la producció.

L'Edat del Bronze (2250-1550 cal. a.n.e.) al sud-est peninsular suposa el desenvolupament d'elits (l'Argar), el que es correlaciona amb la proliferació d'ornaments metàl·lics (coure, plata, or, bronze) i d'armes com símbols d'estatus. Al nord-est, al voltant de 2700 a.n.e., emergeix el fenomen campaniforme. Aquestes comunitats inhumen a un menor nombre de persones que les seves antecessores i aquestes són individualitzades dins de les tombes comunals. Els grups campaniformes produïen eines i armes metàl·liques, que apareixen dipositades, amb evidències d'ús prolongat, en contextos funeraris relacionades amb individus específics. Això mostra una funció tant utilitària com possiblement identitària. La major importància aparent del metall per a les comunitats campaniformes que per als seus antecessors pot relacionar-se amb el començament incipient de l'estratificació social. No obstant això, un total desenvolupament de poder elitici en aquesta àrea no tindrà lloc fins a l'Edat del Ferro; la producció de coure es manté en una escala reduïda durant segles. Finalment, al sud de França, tot i els canvis observats a Cabrières, la producció i consum de metall es redueix en el Llenguadoc durant el final del III mil·lenni a.n.e. La producció es transfereix a l'àrea de Hautes-Alpes fins al II mil·lenni. Això es pot relacionar amb certs canvis socials al nord d'Itàlia.

3. Evidències de metal·lúrgia de gresol al nord-est peninsular

Les evidències de mineria durant el III mil·lenni es concentren en els districtes miners del Montsant (incloent les mines de la Solana del Bepo i La Turquesa) i del Molar-Bellmunt-Falset. S'han trobat evidències de metal·lúrgia extractiva la Cova Joan d'Ós (Tartareu), la Cova del Frare (Matadepera), la Cova Freda de Montsant (Collbató), Vapor Gorina (Sabadell), la Balma del Duc (Montblanc), la Cova del Buldó (Montblanc), la Cova Cartanyà (Vilavert), la Cova de l'Heura (Ulldemolins), la Cova de Porta Lloret (Siurana) i la Cova Josefina d'Escornalbou (Ruidecanyes).

Els materials trobats i els seus contextos denoten una metal·lúrgia de coure rudimentària, domèstica i esporàdica. No hi ha especialització; es porta a terme en hàbitats temporals en cova (excepte a Vapor Gorina), la qual cosa contrasta amb els contextos oberts del sud de la península ibèrica i França. En aquestes coves també es duien a terme inhumacions, però aquests enterraments no es relacionen amb els contextos de producció-hàbitat (excepte a la Cova del Buldó). El nivell II.5 de la Bauma i el context de la Cova de l'Heura destaquen per tractar-se d'àrees dedicades exclusivament a la producció de metall.

4. Materials analitzats i els seus contextos arqueològics

S'han analitzat set fragments de vasos metal·lúrgics campaniformes escorificats. També es publiquen els resultats d'unes metalografies realitzades per S. Rovira anys enrere en tres gresols més. Tres dels deu vasos estudiats presenten decoracions incises. Tots els vasos provenen de cinc nivells diferents datats per ^{14}C. Entre els nivells mostrejats hi ha diferents contextos d'habitació i un context de producció metal·lúrgica (nivell II.5). Totes les mostres analitzades pertanyen a l'horitzó Calcolític Campaniforme del III mil·lenni a.n.e. La informació relacionada amb els contextos arqueològics es pot trobar a Alcalde *et al.* [1–4] i Soriano [5].

5. Mètodes

Després de les anàlisis de fluorescència de raigs X (pXRF) en 32 fragments de vasos metal·lúrgics, sis fragments (H12, E13, G11, F12, F11 i G10) es van mostrejar i van preparar per a la seva anàlisi sota microscòpia òptica i electrònica de rastreig (SEM-EDS). També es van realitzar anàlisis d'isòtops de plom (MC-ICP-MS) en cinc mostres (H12, E13, E11, F11 i G10).

6. Resultats

6.1 Caracterització de les ceràmiques tècniques.

La mateixa argila va ser usada per a la manufactura de tots els vasos metal·lúrgics: un alumini-silicat amb FeO, K_2O i CaO i presència menor de MgO, Na_2O i TiO_2. Les inclusions minerals en la ceràmica són diverses (quarsos, feldespats, aluminosilicats de Fe i minerals de Ti i Zr), de mida variable i formes angulars o subangulars. G10 i H12 presenten inclusions orgàniques. Probablement, les inclusions minerals i orgàniques són components naturals de l'argila.

6.2 Caracterització de les operacions metal·lúrgiques.

Gran part de la capa escorificada és ceràmica fosa. S'observen diferències pel que fa a la ceràmica en les quantitats de MgO, P_2O_5, CaO i CuO. Les capes d'escòria de G10, G11 i F12 són més gruixudes i estan enriquides en CaO, MgO i P_2O_5. H12 té una capa d'escòria gruixuda però menor enriquiment en CaO. L'enriquiment de CaO i MgO pot indicar l'ús de minerals amb ganga calcítica o dolomítica. L'enriquiment en P_2O_5 està relacionat amb el combustible utilitzat o amb la ganga. E13 i F11 tenen unes capes d'escòria més fina; no estan tan enriquides en CaO, però tenen un lleuger enriquiment de K_2O pel que fa a la ceràmica, possiblement derivat de el combustible utilitzat.

6.2.1. Operacions metal·lúrgiques amb càrregues riques en Ca

F12, G10, G11 i H12 representen operacions de reducció de menes de coure riques en Ca (calcita/dolomita). Presenten un enriquiment general en CaO i MgO, i neosilicats (freqüentment anortita) rics en aquests elements. Totes les mostres contenen delafossita. G11 i H12 contenen microestructures que es poden relacionar amb relictes de mena. Els alts nivells de FeO d'alguns neosilicats i àrees de l'escòria es relacionen amb la descomposició d'alguns minerals de ferro de la pasta ceràmica. Les inclusions metàl·liques analitzades són de coure, amb impureses de Fe (G11, G10, H12), As (G11, G10, H12), Ag i Sn (G11), en alguns casos. Les metalografies prèviament realitzades per S. Rovira (PA6326, PA6325 i PA6327) mostren característiques similars a les noves mostres analitzades, podent-les integrar en el mateix tipus d'operacions.

6.2.2. Operacions metal·lúrgiques amb càrregues pobres en Ca

E13 i F11 no contenen evidències contundents de reducció, però és probable que representin aquesta pràctica. E13 conté aluminosilicats de Fe secundaris, delafossita i anortita. La seva presència és coherent amb operacions de reducció, però mai apareixen agrupats, de manera que no es poden relacionar amb relictes de mena. E13 presenta exsolucions de cuprita al voltant de gotes metàl·liques que podrien indicar una re-oxidació. El Pb present a la matriu vítria denota que la càrrega era rica en aquest element. F11 conté una capa d'escòria enriquida en FeO, que deriva, possiblement, de la descomposició de minerals de Fe de la pasta ceràmica. Les inclusions metàl·liques/oxidades analitzades en les dues mostres són de coure amb petites impureses de Fe i As.

6.3 Isòtops de plom

H12 es pot relacionar amb la mina de la Solana del Bepo; G10 amb la de la Turquesa. Això últim és coherent amb la presència d'As en aquesta mostra, tot i que la ràtio ^{207}Pb / ^{206}Pb s'allunya una mica. Es poden trobar minerals rics en Ca en l'entorn d'aquestes mines. F11 es pot relacionar amb la mina de Les Ferreres, que no compta amb minerals de Ca en el seu entorn immediat. E13 i E11 no concorden amb les mineralitzacions caracteritzades del nord-est, els Pirineus i França. Els seus valors segueixen la mateixa tendència que altres mostres del nord-est, per la qual cosa seria possible relacionar-les amb altres recursos regionals no caracteritzats de moment.

7. Discussió i conclusions

A la Bauma es van utilitzar cinc menes, una de la mina de les Ferreres (a 18 km de la Bauma), dues de les mines de la Solana del Bepo i la Turquesa respectivament (a 200km ambdues), una quarta no caracteritzada però possiblement de la regió, i una cinquena polimetàl·lica (Cu-Sn) caracteritzada en un estudi anterior, probablement de les mines de les Ferreres o Can Manera.

Els gresols analitzats es van utilitzar per reduir coure d'acord amb la tradició Ibèrica: ús de vasos metal·lúrgics, menes oxídiques i condicions reductores moderades. Tot i la connexió tecnològica amb el sud de la península

ibèrica, el nord-est és una esfera cultural separada. No està clar que les ceràmiques no decorades es fabriquessin amb propòsits metal·lúrgics. Aquestes ceràmiques estan fetes amb la mateixa argila que les ceràmiques comunes decorades, que es van reutilitzar per produir metall. Les inclusions orgàniques i minerals són components naturals de l'argila.

El coure, al Calcolític, era valorat per les seves característiques utilitàries. Això es reflecteix en l'organització de la producció, i en la manufactura d'eines-arma. La demanda de coure era generalment baixa i la producció de caràcter domèstic. El nivell II.5 reflecteix un moment puntual de major demanda. Aquesta adaptabilitat de la producció és consistent amb una consideració utilitària coure, ja que un ús funerari/simbòlic faria difícil justificar episodis com el del nivell II.5.

El coure tenia un incipient valor social: objectes molt usats es dipositen en contextos funeraris, la qual cosa es pot relacionar amb la manca d'evidències de reciclatge. L'àmplia disponibilitat de minerals de coure permetia produir més metall i que els objectes adquirissin un paper simbòlic en les tombes.

L'explotació de diverses menes contemporàniament es dóna en diferents nivells d'ocupació. Un canvi de funció del jaciment (domèstica *vs.* producció) no implicava la reorganització de la feina més enllà de multiplicar les operacions. Existia una gran versatilitat per adquirir minerals que suggereix un coneixement dels minerals locals i un manteniment de xarxes regionals. Aquesta flexibilitat asseguraria l'accés als recursos necessaris, facilitant el desenvolupament d'una metal·lúrgia de caràcter principalment utilitari. Els recursos minerals esmentats van ser explotats per diferents comunitats calcolítiques alhora.

Durant el II mil·lenni a.n.e., al sud de la península ibèrica, es desenvolupa el poder elitici, contribuint a incrementar el paper simbòlic del metall. Alhora, la producció de metall al Llenguadoc desapareix. Al nord-est, el procés d'estratificació social s'estén en el temps i el coure competeix amb altres matèries primeres en els àmbits simbòlic i utilitari. Certs canvis en el valor del metall s'observen al voltant del 1600 a.n.e., però no és fins a l'Edat del Ferro I (750 / 650-550 a.n.e.) quan es desenvolupen cabdillatges a la vall del Segre-Cinca. D'aquesta manera, el sud de la península ibèrica, el nord-est i el sud de França mostren trajectòries diferents cap a la complexitat social, en les què el paper del coure difereix. És fonamental atendre a factors específics socials, polítics i mediambientals per explicar els canvis sòcio-tecnològic al llarg del temps. Aquest article demostra que és possible contribuir a aquesta narrativa a través de l'anàlisi de restos de producció.

Bibliografia (es remet al lector/lectora a l'article original per a una llista més complerta)

1. Alcalde G, Molist M, Montero I, Planagumà L, Tled A. Producciones metalúrgicas en el nordeste de la Península Ibérica durante el III milenio cal. a.C.: el taller de la Bauma del Serrat del Pont (Tortellà, Girona). Trabajos de Prehistoria. 1998;55: 81–100.

2. Alcalde G, Molist M, Saña M, Toledo A. Procès d'ocupació de la Bauma del Serrat del Pont (La Garrotxa) entre el 2900 y el 1450 cal AC. Museu Comarcal de la Garrotxa; 1997.

3. Alcalde G, Molist M, Toledo A. La Bauma del Serrat del Pont. Memòria de les campanyes d'excavació 1991-1994. 1994.

4. Alcalde G, Molist M, Toledo I Mur A, Caravaca J, Codina D. La Bauma del Serrat del Pont, Tortellà, La Garrotxa: un taller de metal·lúrgia del coure d'ara fa 4000 anys. Annals de l'Institut d'Estudis Gironins. 1994;XXXIII: 43–48.

5. Soriano Llopis I. Producción Metalúrgica Prehistórica en el Nordeste de la Península Ibérica (Mediados del IV-II Milenio cal. ANE). Aportaciones Cronoculturales, Tecnológicas y Funcionales. Vol.I. PhD thesis, Universitat Autònoma de Barcelona. 2010.
